# A Low-Cost Wearable Multimodal Brain Signal Acquisition System Integrating EEG and fNIRS for Depression Detection

**DOI:** 10.3390/bios16090478

**Published:** 2026-08-31

**Authors:** Zihan Fei, Hao Li, Zhongyuan Ying, Xingxing Li, Yuezhou Zhang, Qizhi Zhao, Bin Lian, Weiming Cai, Jialin Cui, Tao Yu, Xianghong Zhao, Shuhao Lv, Zhengxiang Yu, Guanxiang Ding, Yuzhou Ying, Yuhang Zhu

**Affiliations:** 1 College of Information Science and Engineering, NingboTech University, Ningbo 315100, China; fzh0328@nbt.edu.cn (Z.F.); leodesksss@nit.zju.edu.cn (H.L.); zqz@nbt.edu.cn (Q.Z.); lianbin_a@163.com (B.L.); caiwm@nit.zju.edu.cn (W.C.); cuijialin@nbt.edu.cn (J.C.); ylxyt@nbt.edu.cn (T.Y.);; 2 The Affiliated Kangning Hospital of Ningbo University, Ningbo 315201, China; xingli0715@163.com; 3 Department of Biostatistics & Health Informatics, Institute of Psychiatry, Psychology and Neuroscience, King’s College London, London WC2R 2LS, UK; yuezhou.zhang@kcl.ac.uk

**Keywords:** wearable brain-imaging devices, conductive rubber material, EEG, multimodal data acquisition, depression detection, fNIRS, VMD

## Abstract

Wearable brain-imaging devices have been developed to meet the growing demand in the healthcare industry for long-term monitoring of brain signals in natural conditions, such as monitoring brain diseases and emotions. However, conventional EEG and fNIRS (functional near-infrared spectroscopy) devices are often expensive, bulky and difficult to operate, making it difficult to monitor patients for long periods in natural conditions. To address these issues, this article proposes a low-cost, portable and multimodal wearable brain signal acquisition scheme. It combines EEG (electroencephalography) and fNIRS to reflect brain activity from different perspectives. In order to make it more wearable, a conductive rubber material is used as the electrode for the EEG. In this study, the corresponding experiments were used to verify the performance of the device. The first is the measurement of internal system noise, which satisfies the data acquisition of EEG and fNIRS at different gain levels. The *α*-rhythm experiment and the SSVEP (steady-state visual evoked potentials) experiment were used to validate the performance of EEG data acquisition. The performance of the fNIRS was verified by measuring changes in cerebral blood oxygen during breath-hold and breathing. In addition, by decomposing the raw fNIRS data with the VMD (variational mode decomposition) algorithm and performing correlation analysis, heart rate information was separated from the data. The performance of the proposed device was validated in the above experiments, confirming the feasibility of the design for multimodal data acquisition and meeting the requirements for portability and wearability. Furthermore, the proposed device was tested with 31 subjects (15 depressive subjects) to detect depression. Experiments proved the effectiveness of the multimodal signals, which outperformed single modal and surpassed EEG by 8.4% and fNIRS by 23.5%.

## 1. Introduction

With the continuous advancement of technology, the size of chips and sensors is constantly shrinking, which makes wearable devices increasingly smaller. The development trend of wearable medical devices is towards portability, miniaturization, and real-time data [1]. Wearable sensors are widely used in the field of health and wellness through non-invasive and high-precision sampling methods [2,3,4]. Wearable medical devices can reflect the physiological data of the human body through sensors onto people’s mobile devices, making it convenient for healthcare professionals to monitor patients [5,6,7]. However, some devices that monitor the brain, such as functional magnetic resonance imaging (fMRI) and magnetoencephalography (MEG), cannot be made into wearable devices due to their own limitations and large size. As for conventional electroencephalogram (EEG) and functional near-infrared spectroscopy (fNIRS) devices, they have a large number of electrodes and are bulky, especially some EEG devices currently available, which have cumbersome pre-data collection operations and are not suitable for long-term monitoring of users. Some brain disorders require long-term tracking of brain signals, but the conventional brain signal monitoring devices do not meet the requirements for collecting signals in natural states for long periods of time, and wearable brain signal monitoring devices are currently still in the experimental stage [8].

Currently, there is still a lot of research on brain–computer interface (BCI) technology using EEG, and some studies have shown that even with few electrodes, brain signals can still reflect brain activity [9,10]. For example, frontal lobe brain signals have been used in research on brain diseases [11]. EEG is currently the most widely researched brain signal, using electrodes to record electrical signals associated with cortical neuron activity. EEG also meets the requirements for convenience and wearability [12]. Low-cost, wireless, convenient, and easy-to-use consumer-grade wearable EEG devices are becoming increasingly attractive to researchers in various fields, and researchers have compared them with medical-grade EEG devices, proving their effectiveness [13,14]. Anisimov et al. reviewed the development of biological amplifiers, and the emergence of integrated analog-digital chips greatly accelerated the development of portable medical devices for recording bioelectric signals [15]. Pavan Varma Tirumani et al. designed a low-noise and low-cost EEG amplifier using ADS8588 for analog-to-digital conversion, which was applied in BCI, epilepsy seizure detection, and real-time clinical testing [16]. G. Sintotskiy proposed a hardware solution for measuring ear-EEG signals, suitable for daily EEG recording and real-time processing [17]. Ju-Chun Hsieh et al. designed hydrogel electrodes for wearable EEG [18], and Shan Han et al. proposed a method for making earplug-type EEG electrodes based on a polydimethylsiloxane (PDMS) substrate, which has good flexibility and excellent biocompatibility, further enhancing the comfort of wearable EEG [19].

Although EEG signals have been used in medical diagnosis for a long time, there are still some problems with single-mode EEG wearable devices [20]. Functional near-infrared spectroscopy (fNIRS) can be used to detect changes in hemodynamic parameters when different functional areas of the brain are active, and it is currently a very promising brain function detection technology [21]. Francis Tsow et al. designed an open-source, fully integrated wireless fNIRS headband system, which uses a single LED for the light source and four detectors, and verified the hardware with classic fNIRS tasks [22]. Alper Bozkurt used a portable near-infrared spectroscopy system for bedside monitoring of the brains of newborns, and the effectiveness of the system was tested at St. Peter’s University Hospital, using only one pair of near-infrared light sources and two detectors [23]. All of these studies indicate that EEG and fNIRS, due to their low cost, convenience, and non-invasive characteristics, can be well designed as wearable devices to meet users’ needs for continuous and long-term monitoring. However, from current research, EEG, although having a higher temporal resolution, has a lower spatial resolution and a lower signal-to-noise ratio, often accompanied by a lot of interference noise (power interference, physiological noise interference, etc.). fNIRS has a certain spatial resolution capability, but its temporal resolution is very low, and its signal changes are much slower compared to EEG. The sources of EEG and fNIRS signals are different, but they have good homogeneity and their own advantages. In order to accurately, comprehensively, and real-time measure the activity of the brain in cognitive processes, integrating EEG and fNIRS is a better brain imaging strategy [8,24,25,26].

**Figure 1:**
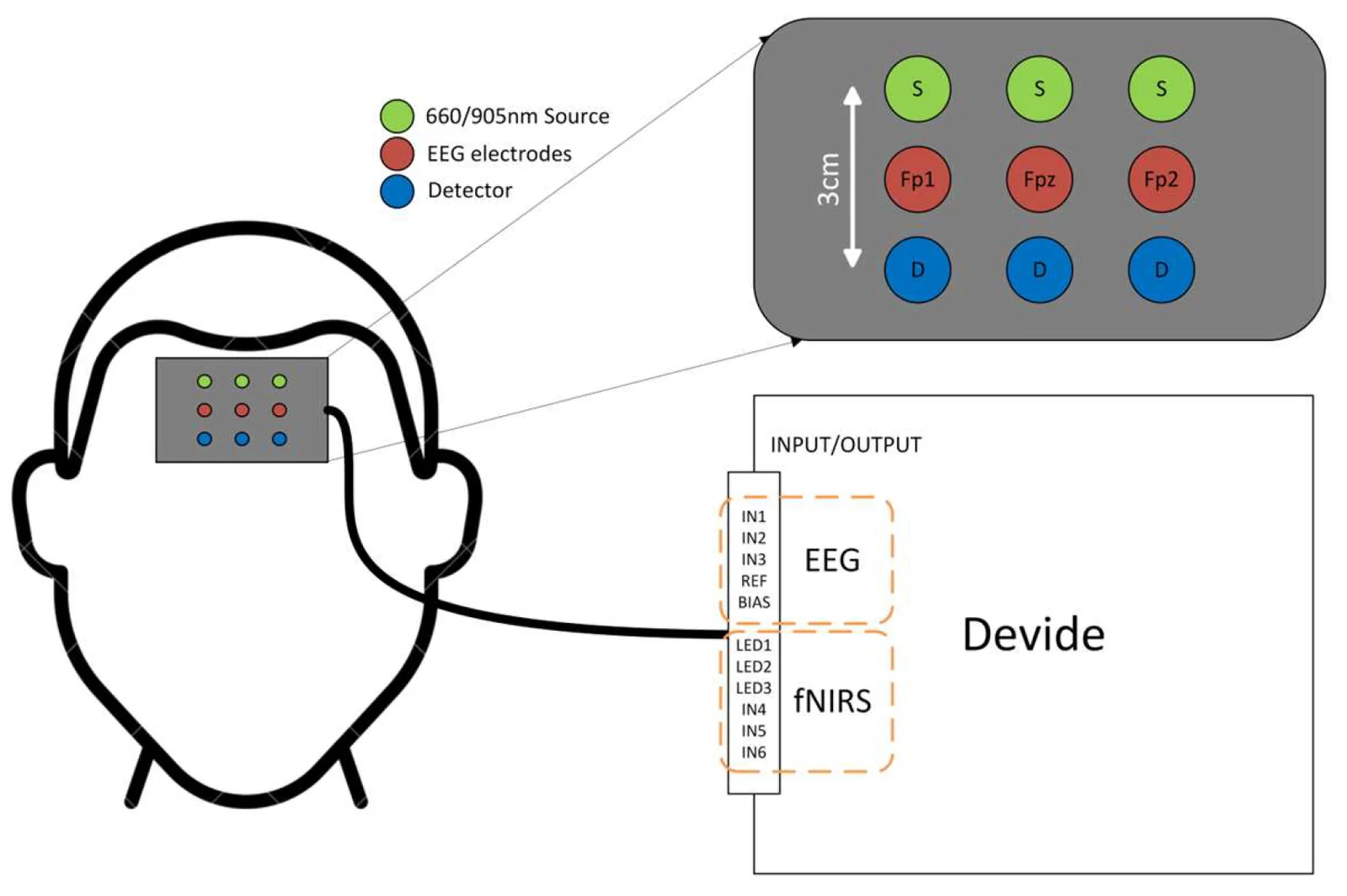
Data acquisition of the device and its processing diagram.

This article proposes and designs a convenient and wearable multimodal (EEG + fNIRS) brain signal acquisition device, considering the significant role of wearable devices in the medical and healthcare fields and the wide application of EEG and near-infrared technology in monitoring brain diseases, emotional monitoring, and assistive therapy. The device can simultaneously collect three channels of EEG signals and three channels of fNIRS signals and is equipped with an accelerometer sensor to filter out invalid data. The system noise was first tested, and then corresponding experiments were designed for the EEG signal acquisition part and the fNIRS signal acquisition part. The feasibility of the EEG part of the device was verified through *α*-rhythm and SSVEP experiments, while the feasibility of fNIRS data acquisition was validated through breath-holding and breathing experiments to monitor changes in brain oxygenation. In the fNIRS experiment, the variational mode decomposition (VMD) algorithm was used to separate the brain oxygenation data and heart rate data from the original fNIRS data, and correlation analysis was performed on the heart rate data. Through these experiments and data analysis, the performance of the device and the feasibility of multimodal data acquisition were preliminarily confirmed, meeting the requirements of convenience and wearability. The data acquisition and processing workflow of the device is illustrated in Figure 1.

## 2. Materials and Methods

### 2.1. System Design

In order to meet the current demands for long-term monitoring of brain signals in a natural state using multiple modalities in the medical field, this article proposes a convenient and wearable EEG-fNIRS signal acquisition device. The device consists of two main functional parts: the collection of electroencephalography (EEG) signals and the collection of near-infrared spectroscopy (fNIRS) signals.

**Figure 2:**
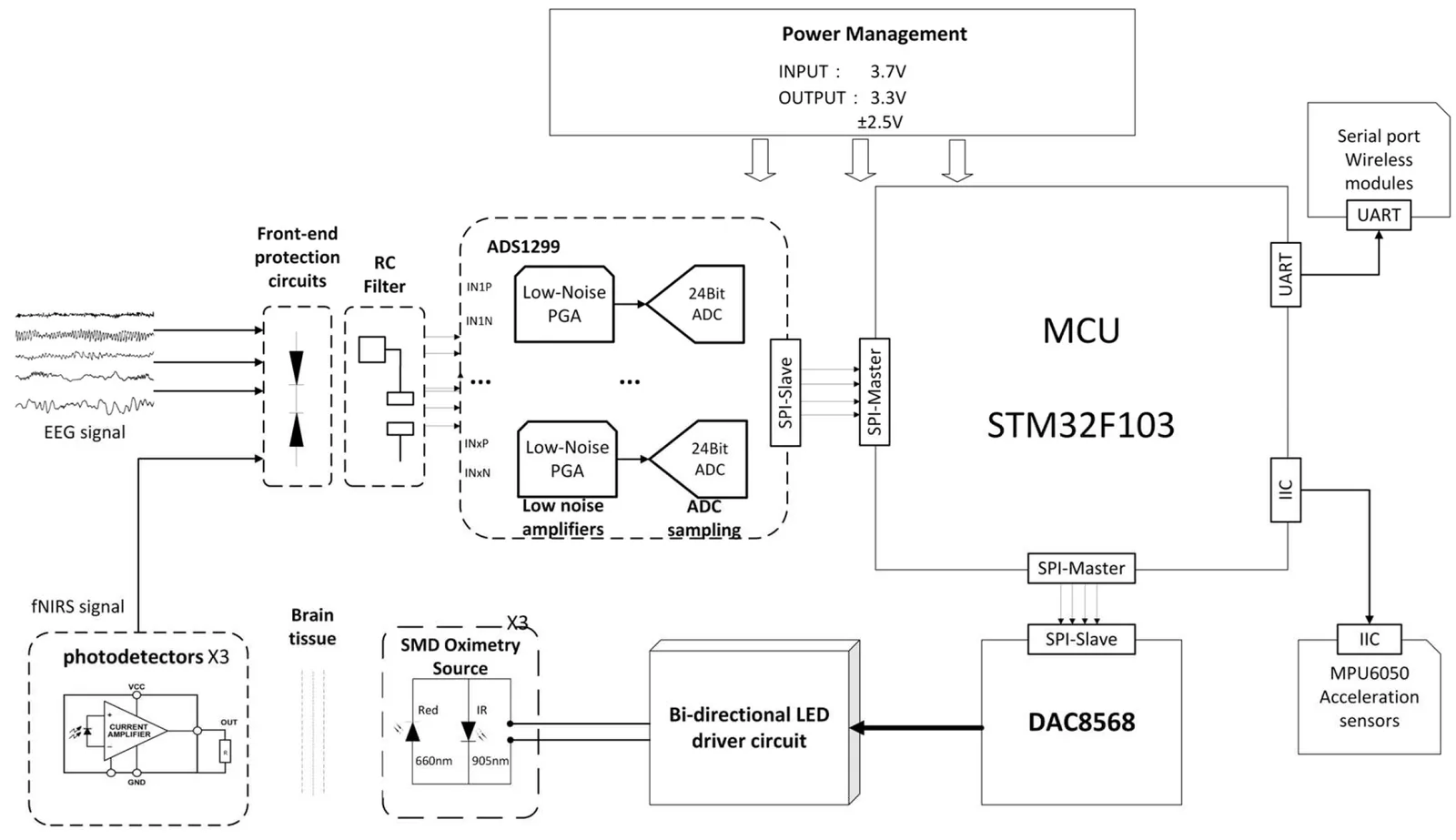
System design block diagram.

The system diagram is shown in Figure 2. The main control MCU (main control unit) uses STMicroelectronics’ (Mascot, Australia) STM32F103 chip, which has rich peripherals and can meet the system’s communication needs. The analog front-end chip for biological signals uses Texas Instruments’ (Dallas, TX, USA) ADS1299, which integrates the functions of programmable amplifiers and analog-to-digital converters. Compared with discrete components, this chip has a smaller volume and is very suitable for wearable devices. After entering the front-end protection module and filtering circuit, the EEG signal is directly converted into a digital signal by the ADS1299 and sent to the main control via the SPI protocol. The fNIRS circuit is divided into two parts, consisting of a dual-wavelength light source driving circuit and a receiver that converts light signals into electrical signals. The main control controls the DAC8568 (Texas Instruments) to output a specified voltage through the SPI protocol and selects different wavelengths of LEDs to work through the LED driving circuit. This DAC has the characteristics of low noise and high precision (16 bit), which can accurately control the current flowing through the LED to achieve a constant current and reduce the interference of current fluctuations on the fNIRS signal.

The device has six data channels (3 EEG + 3 fNIRS), all of which are collected by the ADS1299 analog front-end chip, with a sampling rate of 250 SPS. After being amplified by a programmable gain amplifier (PGA) and converted to digital signals by an internal ADC, the signals are finally transmitted to the MCU. Considering the need for long-term monitoring of brain signals in natural states, we added an MPU6050 accelerometer sensor (TDK, San Jose, CA, USA) to the above circuit to filter out invalid data collected during intense physical activities. During data collection, the device needs to be in a relatively calm state to obtain valid data. When the value of the accelerometer sensor is very large, it can be roughly assumed that the subject is in a state of intense activity. In subsequent signal processing, useless data can be discarded by comparing the accelerometer sensor signal. Our accelerometer screening was performed as follows: based on the triaxial acceleration synchronously collected by the MPU6050 sensor, we first computed the resultant acceleration magnitude

$$SMV(t)=\sqrt{a_{x}^{2}+a_{y}^{2}+a_{z}^{2}}$$

and then normalized it to a Z-score:

$$z(t)=\frac{SMV(t)-median(SMV)}{1.4826-MAD(SMV)}$$

where MAD denotes the median absolute deviation. A sample was classified as stationary only if both conditions were met: |z(t)|<2.5 and the 1 s sliding standard deviation $\sigma_{z}(t)<2.5$.

After sample-wise classification, we applied a 0.5 s dilation to both sides of each movement segment to avoid onset/offset artifacts, merged segments shorter than 3.0 s into adjacent classes, and finally used run-length encoding to obtain continuous stationary EEG segments.

The device is also equipped with a wireless module, and the MCU can send serial data wirelessly to a PC’s host computer, which conforms to the design of wearable devices.

### 2.2. ADS1299 Analog Front-End

The ADS1299 analog front-end chip has 8 channels of low-noise, high-resolution synchronous sampling Δ-Σ analog-to-digital converters, built-in programmable gain amplifiers (PGA), input multiplexers (MUX), internal reference voltages, clock oscillators, bias amplifiers, and internal test sources, as well as lead-off detection circuits, providing all the commonly used functions required for high-precision electrical signal acquisition. The internal device noise of the chip is less than 1 µV, the maximum gain of the programmable amplifier can reach 24, and the ADC has a precision of 24 bits. The data sampling rate supports a maximum of 16 k SPS.

In this design application, the MUX module inside the chip allows each channel to independently set the parameters of the PGA, with adjustable gain, to meet the requirements of simultaneous EEG and NIRS signal acquisition. EEG signals are usually in the µV range, while the photodetector electrical signals of fNIRS are in the mV range, so it is necessary to increase the gain of the EEG channel as much as possible, while for voltages in the mV range, to prevent saturation when voltage quantization occurs, a no-gain setting (i.e., Gain = 1) is typically used.

### 2.3. EEG Acquisition

**Figure 3:**
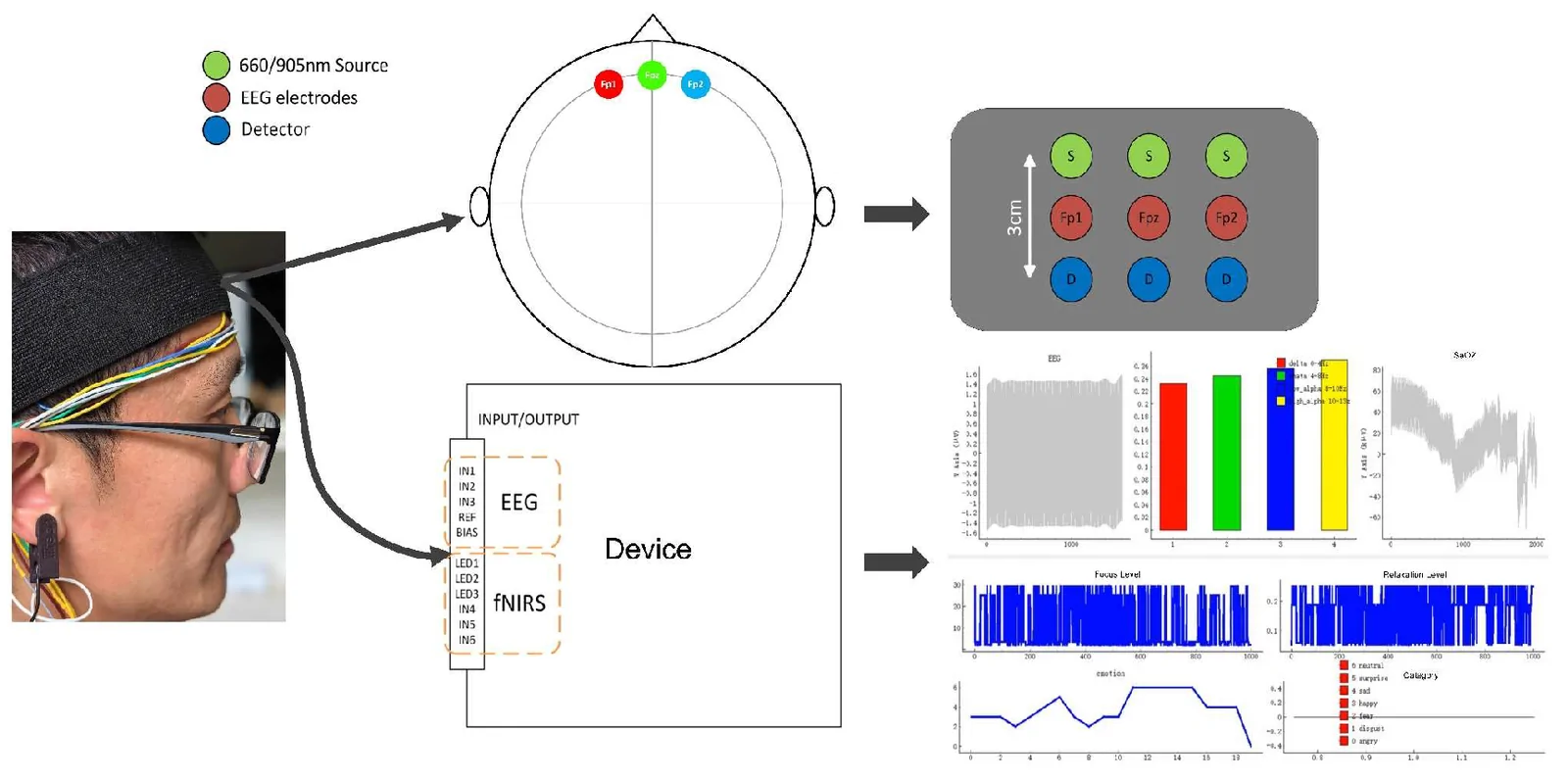
Schematic diagram of EEG electrode and fNIRS positions and wiring.

The wiring diagram for measuring EEG is shown in Figure 3, and the measurement locations are selected at three electrode sites on the forehead, Fp1, Fpz, and Fp2, in the EEG 10–20 international standard electrode placement system. Our research team is particularly interested in the diagnosis and treatment of mental disorders. Studies have shown that the frontal lobe of the brain is primarily responsible for various complex cognitive functions. Brain signals in the front part of the brain have great potential to serve as biological markers for the diagnosis of depression [27,28,29], and these three frontal positions are hairless areas, allowing the electrodes to fully contact the skin, reducing impedance, and making it easier for users to wear. In fact, two different electrode configurations were used for two different purposes. The eyes-open/eyes-closed and SSVEP experiments were designed as functional validation experiments for the EEG amplification and acquisition circuitry. During the SSVEP experiment, conventional Ag/AgCl electrodes suitable for hair-bearing scalps were placed over the occipital region, where visually evoked responses are expected to be strongest. The frontal flexible electrodes were not used for the SSVEP experiment, which distinguishes the frontal flexible-electrode configuration used for depression monitoring from the occipital Ag/AgCl electrode configuration used for SSVEP validation. In addition, it is necessary to connect the reference electrode and the bias electrode to the human body, and this device connects these two electrodes to the left and right ears, respectively. The function of the reference electrode is to set the zero potential, and the potential difference reflected by the EEG is relative to the reference electrode. The function of the bias electrode is to cancel the common mode interference in the EEG system caused by power lines and other sources. A negative feedback loop is created by driving the subject with an anti-phase common mode signal, and the common mode is restricted to a narrow range according to the loop gain of the negative feedback loop.

### 2.4. fNIRS Acquisition

**Figure 4:**
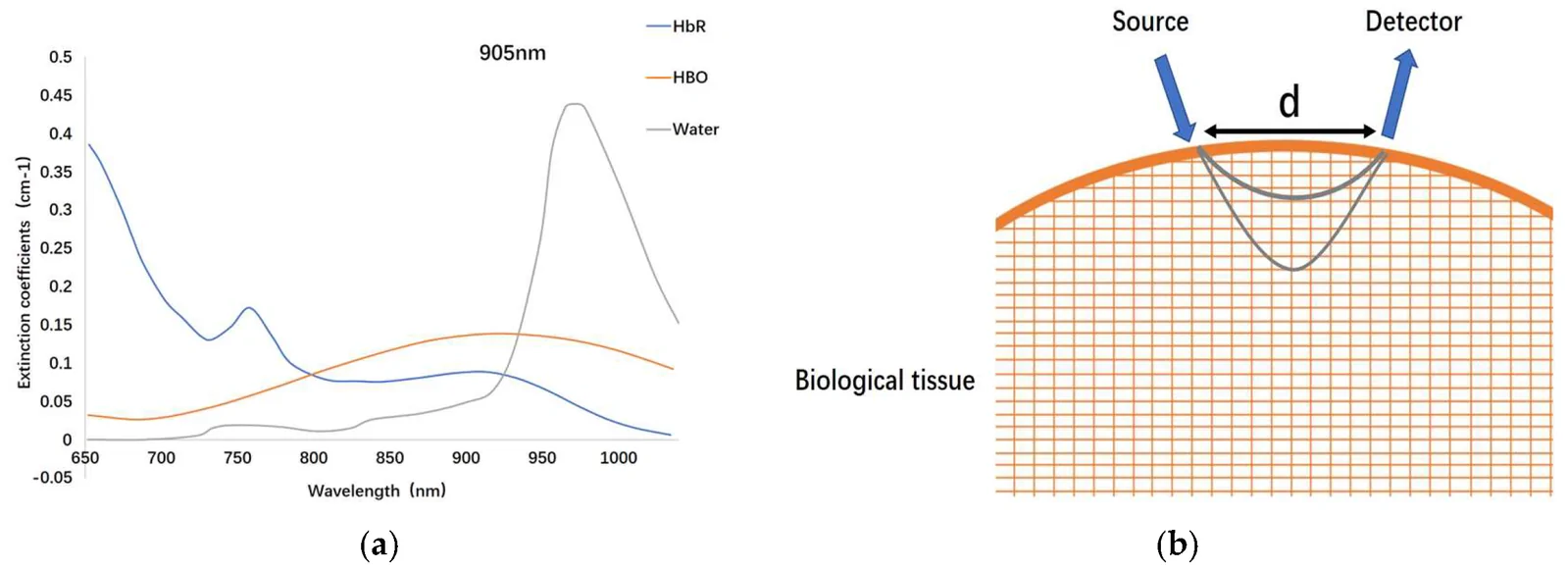
(**a**) Extinction coefficients of oxyhemoglobin, deoxyhemoglobin and water at 650–1100 nm. (**b**) Schematic diagram of the position of the light source and detector and the light path of light propagation through the tissue.

The principle of fNIRS is based on the differential absorption characteristics of oxyhemoglobin (HbO) and deoxyhemoglobin (HbR) in brain tissue at different wavelengths of light. By choosing different wavelengths of near-infrared light to penetrate the brain, the near-infrared light is absorbed and attenuated by HbR and HbO in brain tissue, and then scattered. The changes in light intensity are detected by photodetectors and processed and analyzed to obtain the changes in the concentrations of HbR and HbO in the brain. The absorption coefficients of oxyhemoglobin, deoxyhemoglobin, and water at 650–1100 nm are shown in Figure 4a.

This device provides three channels for near-infrared data acquisition, consisting of an fNIRS light source system and detectors, arranged as shown in Figure 3. By irradiating different wavelengths and intensities of light into the brain tissue, the device measures the brain using the characteristic that different substances in brain tissue have different absorption rates for different wavelengths.

Figure 4b shows the optical pathway of light propagation inside the human brain, which is the result of scattering and reflection of light after entering the head. The distance between the light source and detector reflects the activity of different depths in the brain. If the distance is too close, the received light may not penetrate the skull and enter the brain. If the distance is too far, the received light signal may be too weak, resulting in a low signal-to-noise ratio. Intervals below 1 cm may only penetrate the skin layer, while intervals exceeding 5 cm, although detecting deeper parts of the brain, may weaken the signal. The distance between the emitter and detector pairs is typically 3 cm [30].

In order to address various problems caused by scattering, Dr. Delpy et al. modified the Beer-Lambert law and proposed the MBLL formula [31], with the expression shown in Equation (1).

(1) $$OD=\ln\frac{I_{inc}}{I_{det}}=DPF\left(\lambda \right)d\mu_{a}+G$$

The variable $I_{inc}$ represents the incident light intensity, $I_{det}$ is the detected light intensity, λ is the wavelength of the light source, G is the light loss parameter associated with scattering, DPF (Differential Path length Factor) is the factor for differential path length, and d is the straight-line path of light. The product of DPF and the transmission distance d can represent the average optical path of photons during transmission, as shown in the banana-shaped area in Figure 4. In general, the parameter G associated with scattering can be assumed to be constant. The rate of attenuation in optical density can be expressed as Equation (2).

(2) $$\Delta OD=\ln\frac{I_{det,1}}{I_{det,2}}=DPF\left(\lambda \right)d\Delta \mu_{a}$$

The optical path loss parameters for $HbO_{2}$ and HbR are brought into the above equation to obtain Equation (3).

(3) $$\Delta OD=DPF\left(\lambda \right)d\left(\varepsilon_{HbO_{2}}\left(\lambda \right)\Delta c_{HbO_{2}}+\varepsilon_{Hb}\left(\lambda \right)\Delta c_{Hb}\right)$$

$$\Delta c_{HbO_{2}}=c_{HbO_{2}}\left(t_{1}\right)-c_{HbO_{2}}\left(t_{0}\right)$$

$$\Delta c_{HbR}=c_{HbR}\left(t_{1}\right)-c_{HbR}\left(t_{0}\right)$$

For example, when using a dual-wavelength light source, the light intensity loss for different wavelengths at the dual-wavelength light source can be obtained from the above equation as Equation (4).

(4) ΔODλ1ΔODλ2=L⋅εHbRλ1,L⋅εHbO2λ1L⋅εHbRλ2,L⋅εHbO2λ2ΔcHbRΔcHbO2

In the equation, ΔOD (λ1) represents the light attenuation at wavelength λ1, εHbR (λ1) and λ1, εHbR (λ2) represent the absorption coefficients of HbR and HbO2, respectively, at wavelength λ1. L=DPF(λ)·d, where d is the straight-line distance between the light source and the detector. DPF is closely related to individual differences and the wavelength of the light source, but in the calculation of relative concentration changes, it can be simplified as a constant since we are discussing the same individual. In this paper, the DPF is 7.49 when the wavelength is 660 nm. For a wavelength of 905 nm, the DPF is set to 6.38. This value is calculated using an empirical formula.

(5) ΔcHbRΔcHbO2=1dDPFλ1⋅εHbRλ1,DPFλ1εHbO2λ1DPFλ2⋅εHbRλ2,DPFλ2εHbO2λ2−1⋅ΔODλ1ΔODλ2

From this, the relative amounts of the hemodynamic parameters of HbR, $HbO_{2}$ can be calculated from the changes in the detected light signal by Equation (5), d being the distance between the detector and the light source, which in this device is 3 cm.

**Figure 5:**
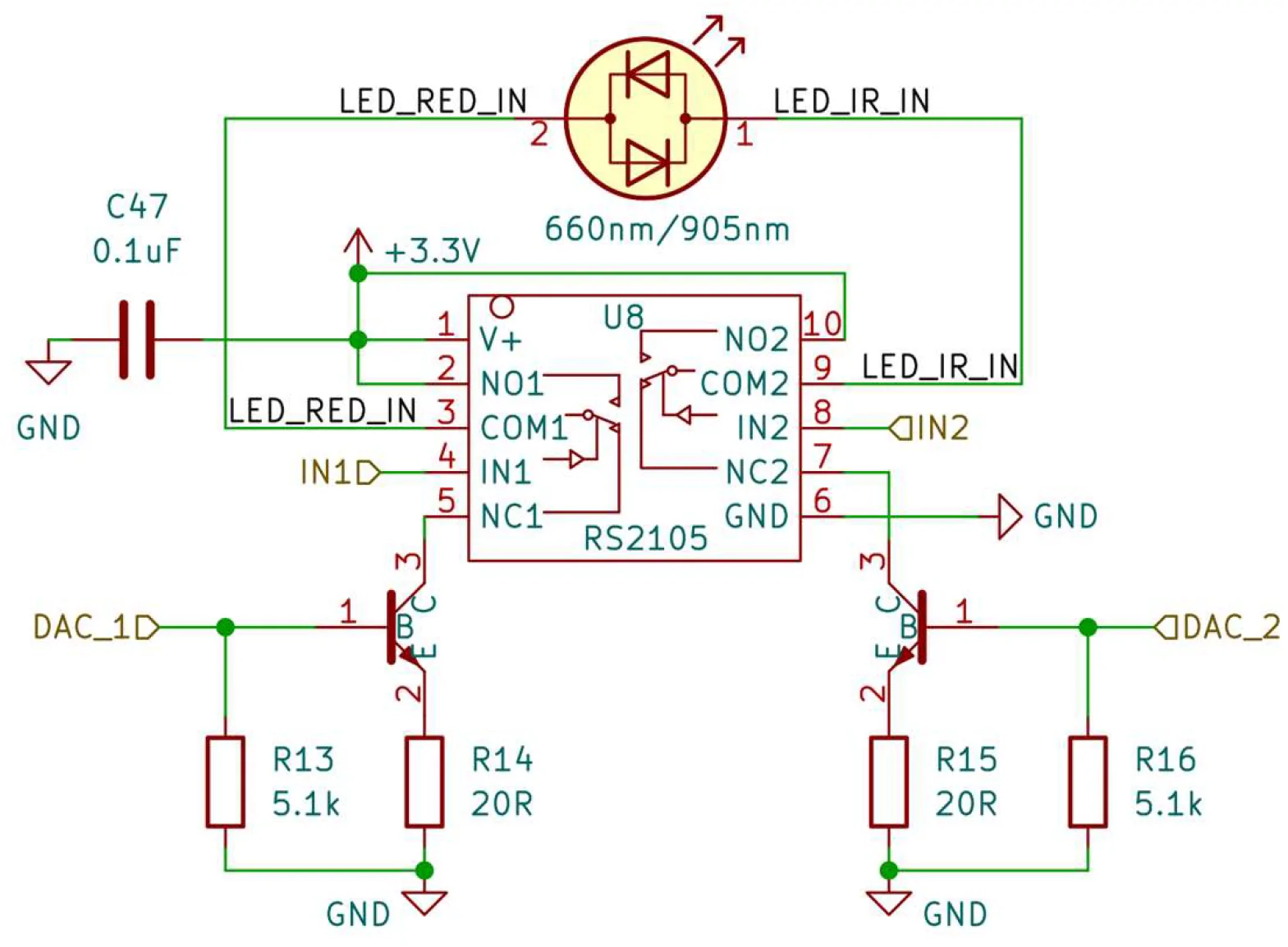
One-channel dual-wavelength LED driver circuit diagram.

Figure 5 shows a dual-wavelength LED driving circuit (660 nm and 905 nm), where each driving circuit consists of two MCU IO ports, two DAC channels, an RS2105 analog switch chip, two transistors, and several peripheral circuits. The two MCU IOs control the RS2105 analog switch, and as shown in the wiring diagram, when COM1 is connected to NC1 and COM2 is connected to NO2, DAC_1 controls the intensity of one of the wavelength LEDs. When COM1 is connected to NO1 and COM2 is connected to NC2, DAC_2 controls the intensity of the other wavelength LED. The 20-ohm resistor in series with the transistor is a current-limiting resistor. In this circuit, the maximum current for each LED does not exceed 100 mA.

### 2.5. Front-End Protection Circuit

**Figure 6:**
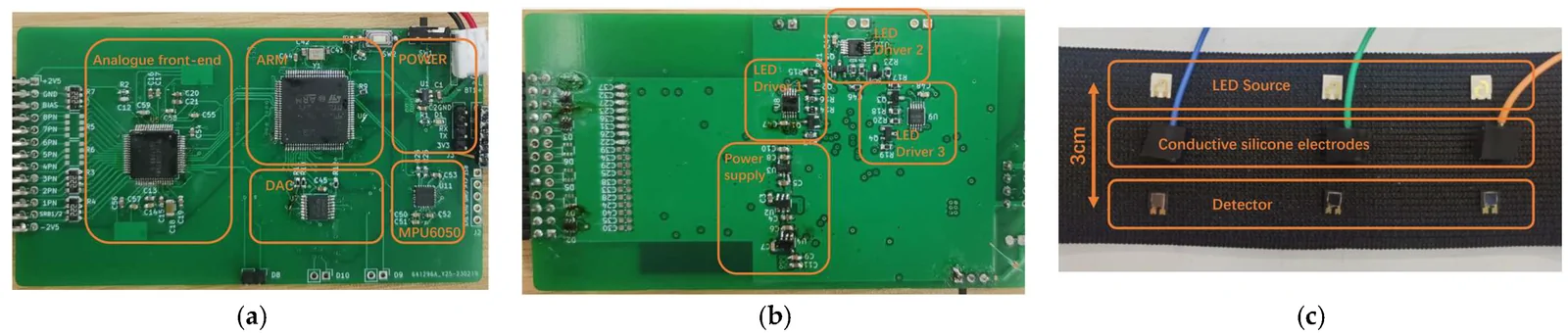
Hardware physical picture display. (**a**) shows the front view of the device, which contains the layout of the analog front-end chip, the master ARM chip, the DAC digital-to-analog converter chip, the MPU6050 accelerometer and part of the power supply chip; (**b**) shows the back view of the device, containing the driver circuit for the three dual wavelength LEDs and part of the power supply chip; (**c**) shows the schematic of the position of EEG conductive rubber electrodes and the position of the fNIRS light sources (660 nm/905 nm) and detectors.

In dry conditions, the human body may generate very high-voltage static electricity. Although static electricity is not harmful to the human body, it can cause significant damage to electronic devices. Considering that the human body surface generates a large amount of static electricity when in contact with the electrode, it may cause unnecessary damage to the signal acquisition module and main control module. Therefore, a front-end protection circuit is designed to prevent this from happening. TPD4E1B06D is a four-channel bidirectional transient voltage suppressor (TVS) from TI. This device has very low leakage current, less than 1 nA, making it suitable for static protection of precision instruments. The schematic diagram of the device’s hardware configuration is shown in Figure 6.

### 2.6. Power Management

The power supply of this device is a 3.7 V lithium battery, with the 4055H chip completing the battery’s charging and discharging functions. The main control requires a 3.3 V digital terminal voltage, so a low dropout linear regulator chip is used to convert the lithium battery voltage to 3.3 V. We use TI’s LP5907MFX-3.3 V chip to complete the voltage conversion. The analog front-end ADS1299 is powered by dual supplies, divided into the analog circuit and digital circuit parts. The analog circuit part needs to provide −2.5 V (AVSS) and +2.5 V (AVDD) voltages, and the digital part needs to provide GND and VCC (+3.3 V). For negative voltage, a power inverter, TI’s LM2664M6 power chip, is used to first convert the voltage to −3.3 V, and then a negative linear regulator chip is used to convert the voltage to −2.5 V. The analog front-end chip has very strict requirements for noise, so the above chips need to use low-noise models.

### 2.7. Data Acquisition Timings

**Figure 7:**
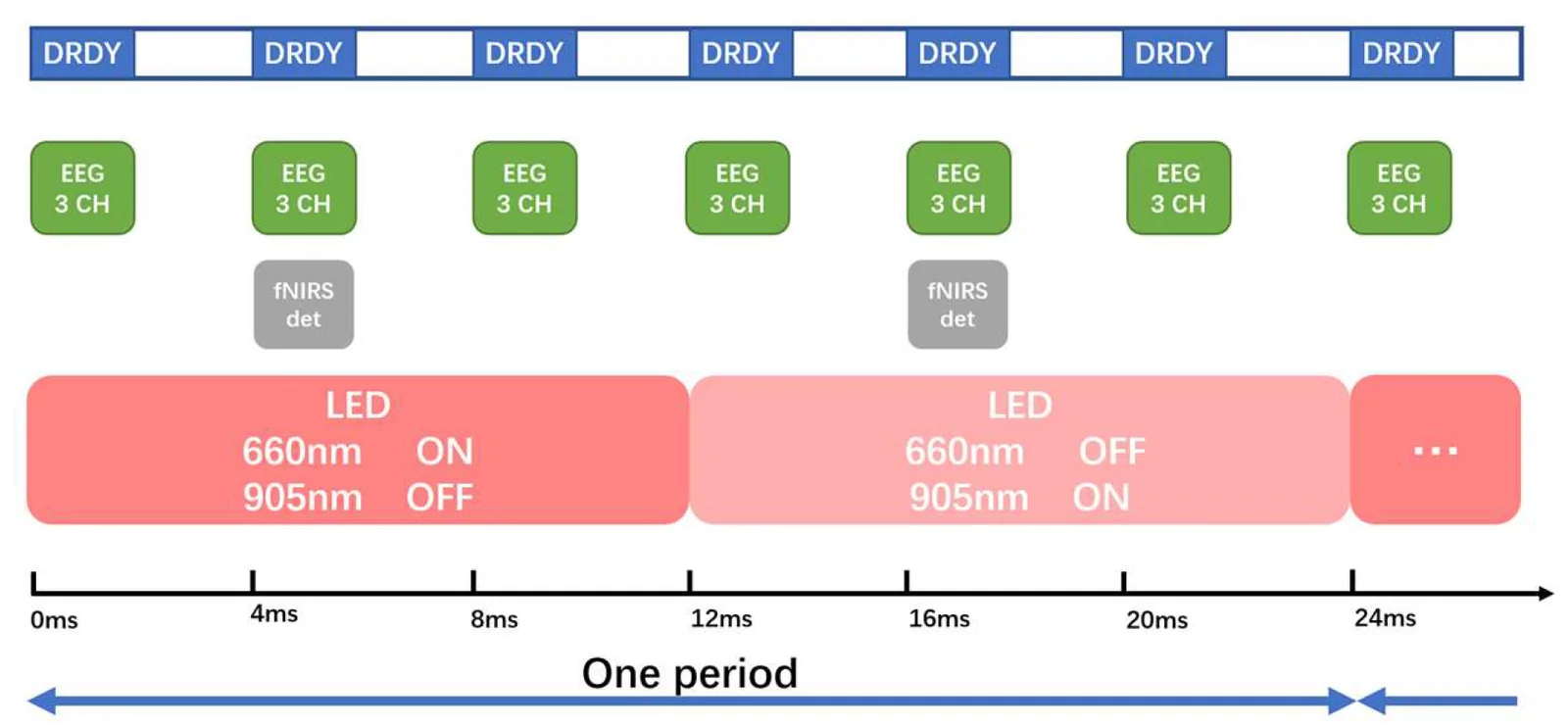
Timing diagram for data acquisition.

Figure 7 shows one period of data acquisition for this device, which includes the data acquisition timing for the EEG and fNIRS and the timing for the dual wavelength LED driver switching. The sampling rate of the ADS1299 analog front-end is 250 SPS, so the minimum interval for data acquisition is 4 ms, and DRDY is the data-ready flag bit, at which point the MCU performs the data reading. The fNIRS LED light source needs to be converted between 660 nm and 905 nm, so the fNIRS data needs to be collected with a stable light source, and the fNIRS data does not need a high sampling rate. Therefore, the two wavelengths of LEDs last for 12 ms each, a period of 24 ms.

### 2.8. PCB Layout

The PCB layout of this design primarily considers signal integrity, as the device pertains to EEG signal monitoring, which tends to be extremely weak. Therefore, it is necessary to guarantee signal routes are short and distant from high-frequency interference sources. When arranging the components, we have ensured all analog chips are far from power conversion chips and wireless modules. Additionally, to safeguard the supply stability of the ADS1299 chip and the DAC8586 chip, we have adopted linear regulation and made certain each device has a good ground connection to minimize power supply noise. The ADS1299 is a high-precision analog front-end, so it is crucial to isolate analog and digital signals. In this design, we have separated analog and digital ground and connected them at suitable places to reduce the influence of digital noise on analog signals. Considering future scalability, the PCB layout can be improved in the following ways: modularizing core functions, for instance, designing EEG signal processing, near-infrared signal processing, data processing, and communication as separate modules. Not only does this facilitate maintenance and upgrading, but it also provides flexibility for the addition of new functions in the future. By utilizing high-density component placement techniques, we can reduce signal transmission distances and reserve some general expansion interfaces, such as SPI, I2C, and UART, during the design phase. This allows for easy addition of new sensors or communication modules in the future. The technical specifications and performance metrics of the device are listed in Table 1.

## 3. Results

### 3.1. Internal Signal Testing and Noise Testing

Inside the ADS1299 analog front-end chip, there is an internal signal source that can be used to evaluate the entire system. The amplitude of the internal signal source is set to (VREFP − VREFN)/2400, where VREFP and VREFN are determined by the internal high-precision voltage reference source and the value of VREFP-VREFN is always 4.5 V, so the amplitude of the internal signal is 1.875 mV.

**Figure 8:**
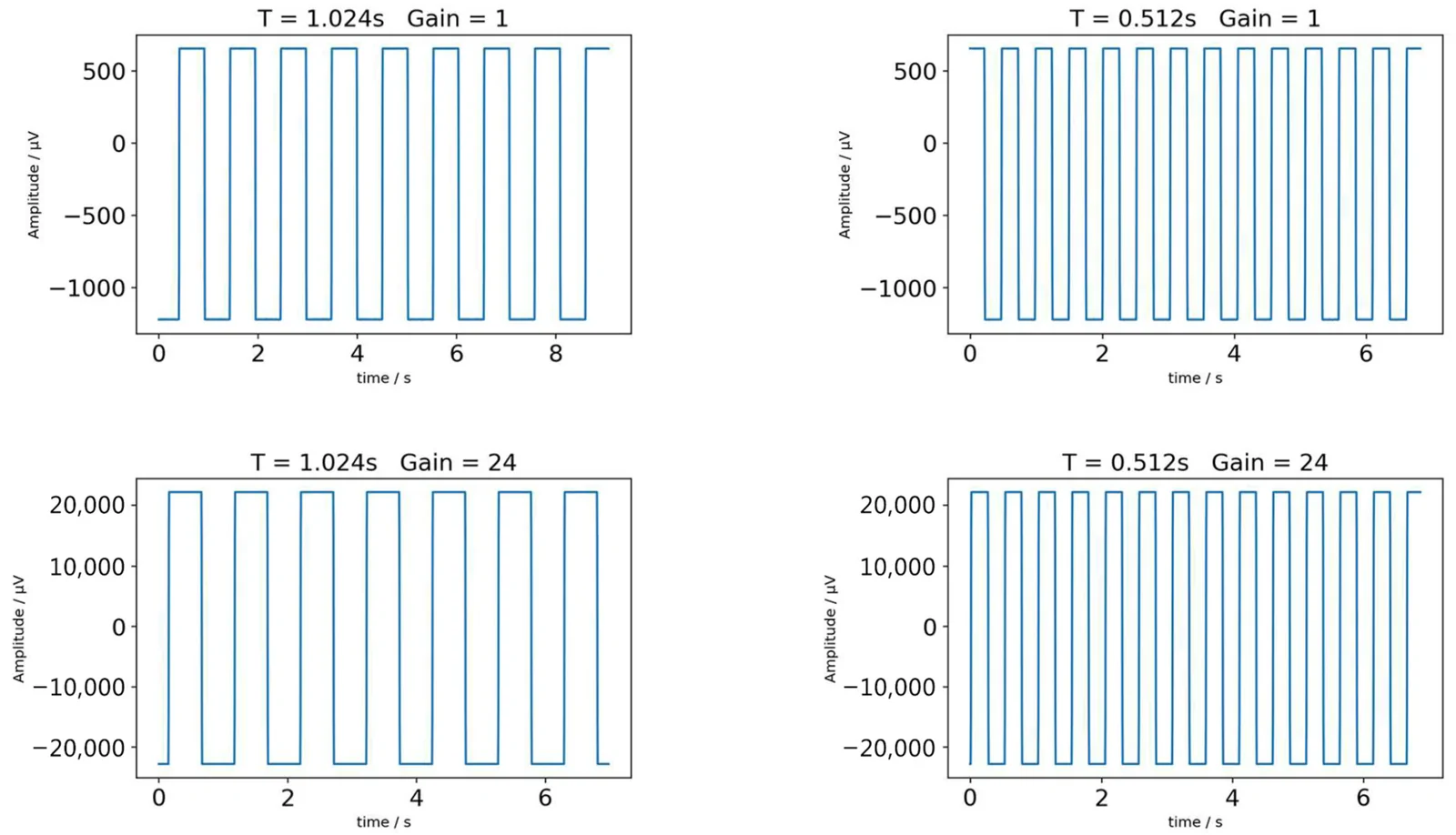
Internal square wave signal testing.

In this experiment, the internal signal source was set to have a period of 1.024 s and 0.512 s, with gains of 1× (no gain) and 24× gain selected. The waveforms collected by the device are shown in Figure 8.

The calculation results of the measurement error of the internal signal square wave are shown in Table 2. It can be seen from the data sheet of the ADS1299 that the accuracy of this test signal is ±2%. The actual measured data error is far less than 2%, which preliminarily verifies the signal acquisition performance of the device.

The amplitude of EEG signals is very weak, and collecting high-quality EEG signals requires that the system’s own noise also be very small. The purpose of this experiment is to analyze the noise. All the electrodes of the EEG acquisition part and the reference electrode are connected and then connected to GND. Under this condition, noise data is collected, and finally, the peak-to-peak value of the noise signal is calculated. This value is divided by the gain to obtain the final noise peak-to-peak value of the system.

(6) $$U_{noise}=\frac{U_{max}-U_{min}}{Gain}$$

The calculation formula for noise is shown in Equation (6), where *Umin* is the maximum value of the collected data, *Umin* is the minimum value of the collected data, and Gain is the gain set by the system. We tested the noise of the system with gains of 1 and 24 and obtained the average noise peak-to-peak value of the system by multiple measurements. The test results of the noise are shown in Table 3. The peak-to-peak value of the noise was 3.31 µV on average with a gain of 1, and 1.53 µV on average with a gain of 24.

### 3.2. EEG Testing Experiment

In the EEG testing experiment, we selected two representative experiments to test our device, namely the alpha rhythm experiment and the steady-state visual evoked potential (SSVEP) experiment. Previous studies have shown that these two experiments can effectively verify the reliability of the signal collection of EEG devices [32,33]. In both experiments, the subjects were in a quiet and comfortable indoor environment, and during data collection, the subjects were instructed to remain as still as possible. The EEG sampling rate was set to 250 Hz.

**Figure 9:**
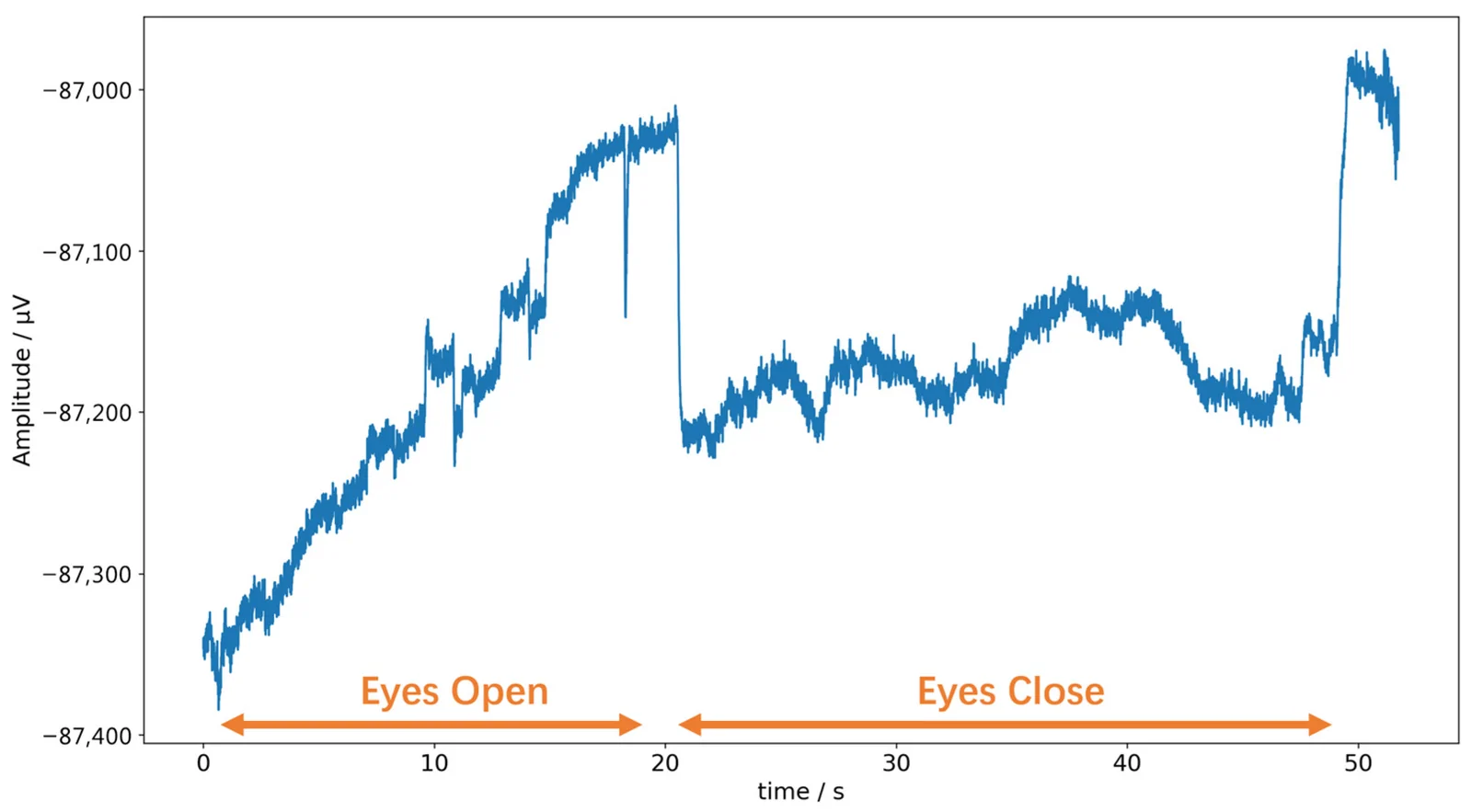
Electroencephalogram with eyes open and eyes closed.

Figure 9 shows the waveform of the raw data collected in the alpha rhythm experiment. In this experiment, we collected data for about 50 s while the subjects were in a resting state with eyes open and eyes closed. It is evident from the time-domain plot that the two states of eyes open and eyes closed can be clearly distinguished.

**Figure 10:**
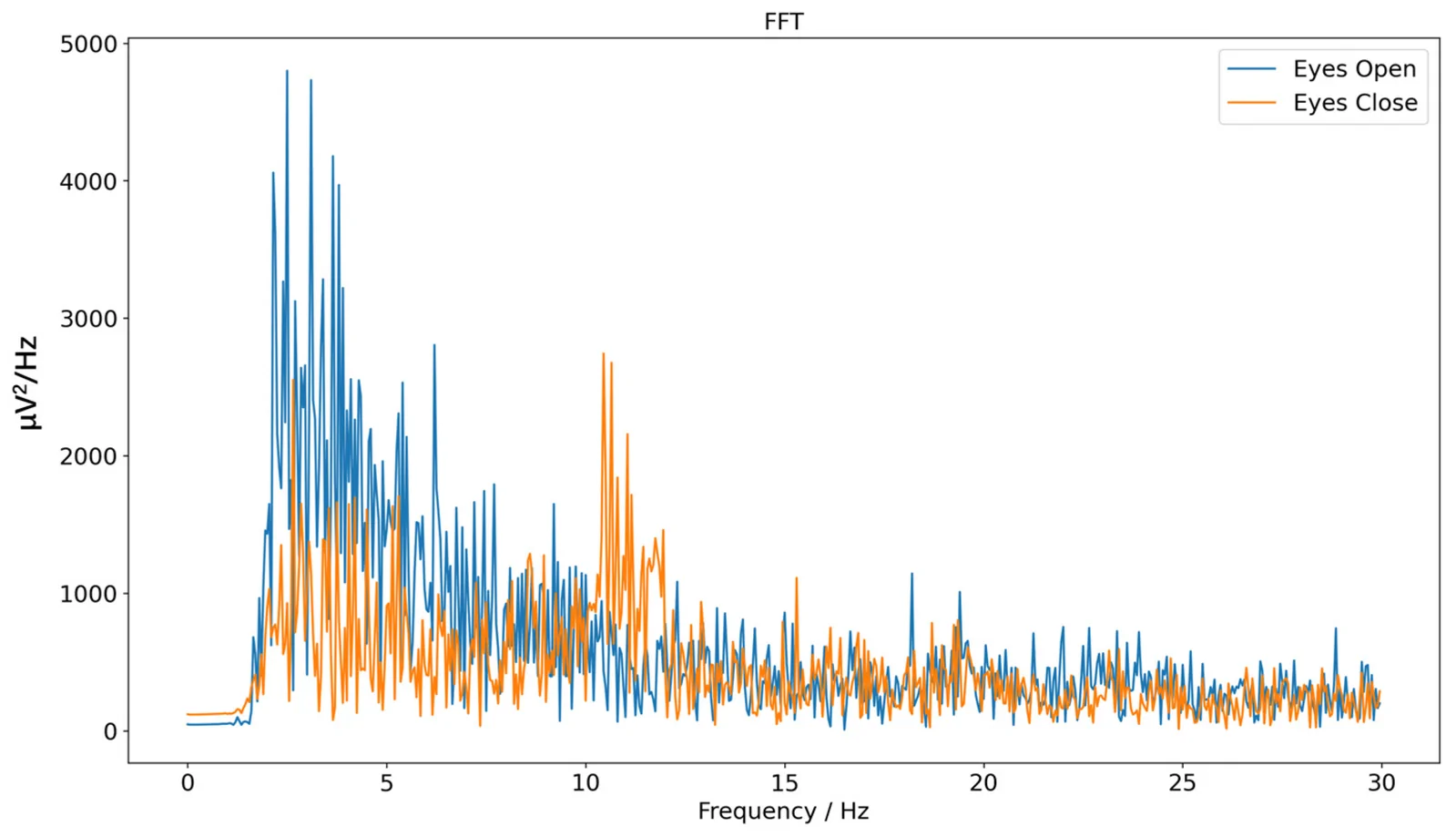
Comparison of the different EEG frequency domains with eyes open and eyes closed.

Alpha waves are one of the basic waveforms of the electroencephalogram (EEG) with a frequency range of 8–13 Hz and an amplitude range of 20–100 µV. They are observed during awake, quiet, and closed-eye states and disappear during eye opening, thinking, and exposure to other stimuli. Figure 10 shows the frequency domain of the original data collected in the alpha wave rhythm experiment after digital filtering and FFT transformation. By comparing the frequency domain plots, it is observed that the maximum spectral energy of the subject in the 2–8 Hz range occurs when the eyes are open and the alpha wave is not obvious. However, when eyes are closed, the spectral energy below 8 Hz decreases, and there is a significant peak in the alpha wave frequency range of 8–13 Hz, indicating that the alpha wave dominates the EEG signal.

**Figure 11:**
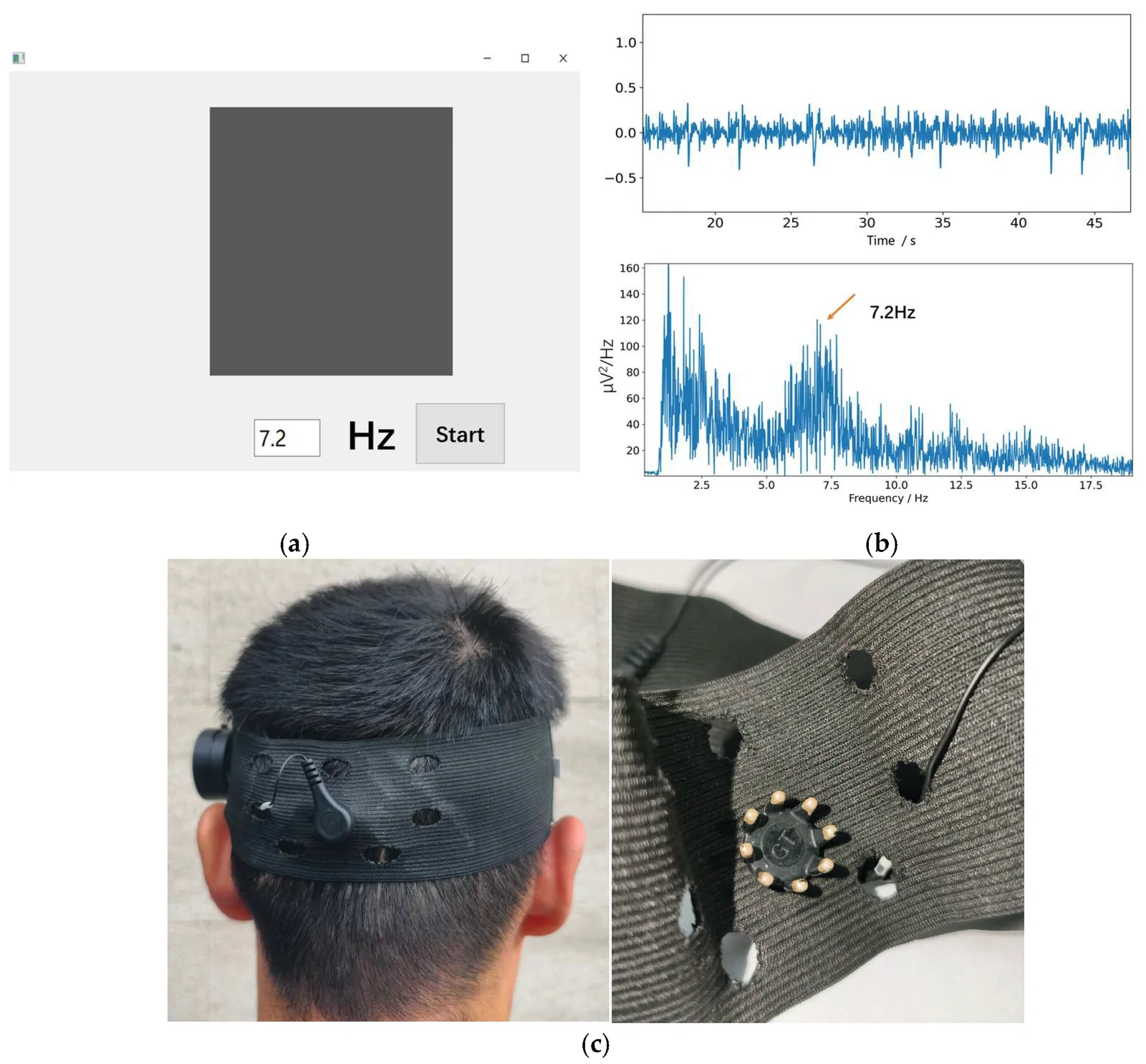
Steady-state visual evoked potential experiments. (**a**) SSVEP program; (**b**) Time and frequency domain plots of EEG signals during SSVEP experiments. (**c**) wearing illustration and electrodes placed on the occipital region.

Steady-State Visual Evoked Potential (SSVEP) is a signal commonly used in brain–computer interface systems. The principle behind it is that when the eyes are exposed to visual stimuli with a fixed frequency of more than 4 Hz, the cortical activity in the brain will be modulated, producing periodic rhythms similar to the stimuli. We designed a program on a computer that can provide a flashing signal with a variable frequency for this experiment, as shown in Figure 11a below. The subjects were required to look at the flashing signal, and we validated the performance of our device by collecting and analyzing their EEG signals.

In the SSVEP experiment, the EEG electrodes were placed on the frontal area to capture the brain’s cortical activity in response to visual stimulation. However, the electrical signals generated by the brain due to visual stimulation are relatively weak in the frontal area, and thus, the environmental requirements are extremely high. The testing environment needs to minimize power frequency interference and light interference. Therefore, all power sources in the testing room need to be turned off, and the upper computer that receives data and the program shown in the previous image need to run on a laptop computer using battery power.

The program was set to flicker at a frequency of 7.2 Hz, and EEG data recording began after the program was launched, with a duration of about 20 s. Figure 11b shows the frequency spectrum of the filtered EEG data collected when the subject watched the flickering program and when they did not. There is a significant difference in the amplitude around 7.2 Hz between the two conditions. When the subject watched the flickering program, the EEG signals collected at the corresponding flickering frequency showed a significant increase in spectral energy. This experiment further confirms the performance of the device in collecting EEG signals.

### 3.3. fNIRS Experiment

**Figure 12:**
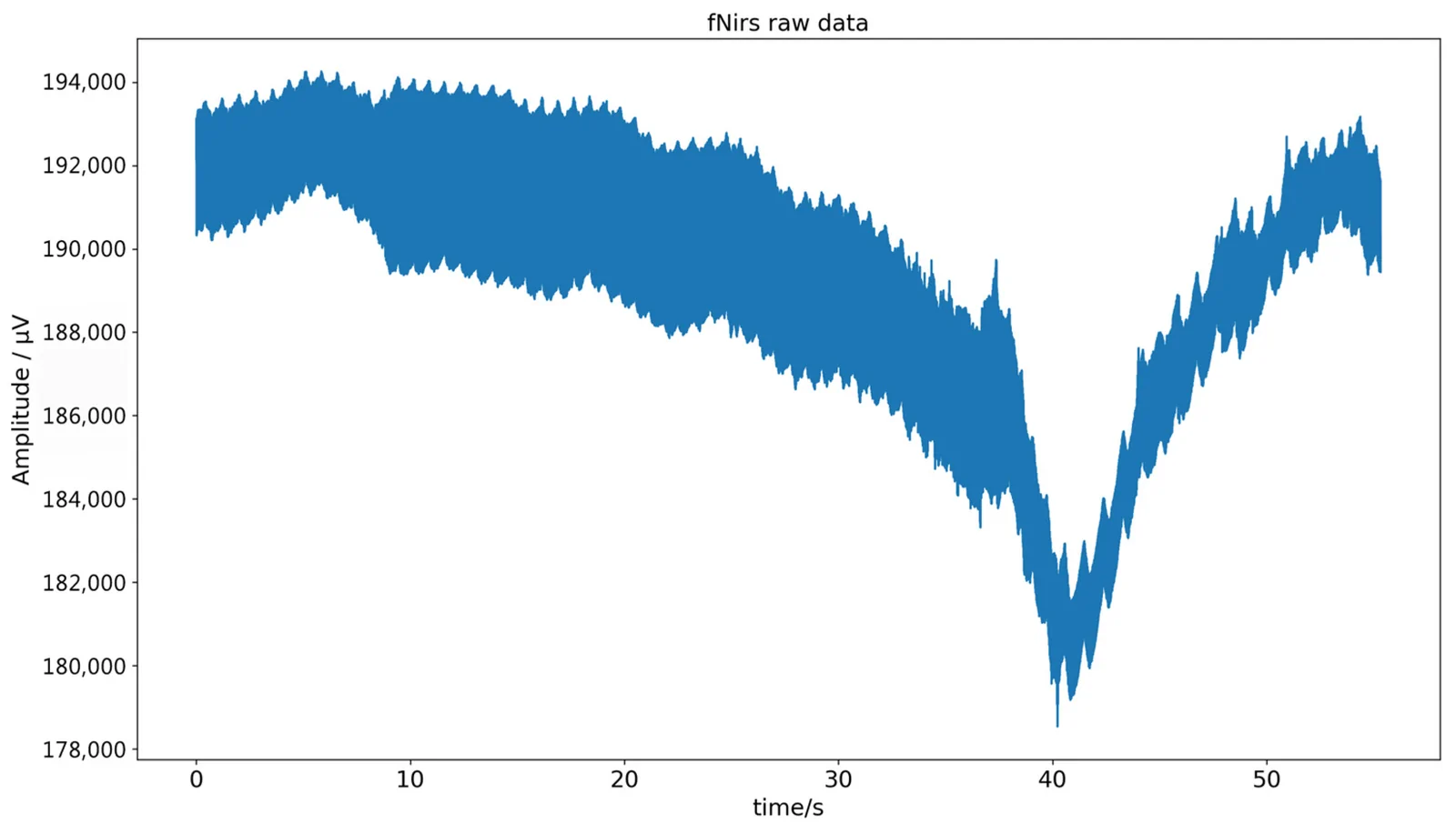
Raw data from fNIRS during breath-holding and breathing.

In order to test the performance of our device in fNIRS, we conducted an experiment to detect changes in brain oxygen levels. In this experiment, the participant was asked to hold their breath, and we analyzed the changes in brain oxygen levels during prolonged breath-holding. The experimental procedure is as follows: Before the test, the participant needs to maintain a stable state of breathing and heart rate and be in a quiet room for measurement. We used a continuous illumination method with a 660 nm wavelength LED to illuminate the frontal brain area, with a distance of 3 cm between the light source and receiver. Then we obtained the data through the receiver and analyzed the changes in brain oxygen levels. When the participant began to hold their breath, data recording began, lasting about one minute. The raw data is shown in Figure 12.

This section chooses to use the Valsalva experiment as the content of the experiment. The Valsalva experiment was first proposed by the Italian anatomist Maria Valsalva in 1704. Its experimental mechanism has been demonstrated through a large amount of research [34], confirming that it can cause changes in the concentration of corresponding blood proteins. Here are the specific steps of the experiment: (1) The subject inhales deeply while remaining calm, then shuts the mouth and nose tightly. (2) With the mouth and nose shut, the subject forcefully exhales while closing the glottis to block the airflow; this action increases the pressure inside the chest, and it is maintained for about 30 s. (3) After the breath-holding period, normal breathing is resumed, and the subject remains calm.

The subject held their breath from 0 s and started deep breathing around 37 s, until their breathing eventually stabilized. At the beginning of breath-holding, there was no obvious change in the received data. Around 20 s, the voltage on the receiver began to gradually decrease. When the subject started inhaling at 37 s, the voltage on the receiver did not immediately increase. Instead, there was a rapid drop period, where the voltage reached its lowest point before quickly rebounding and gradually rising back to its initial state.

**Figure 13:**
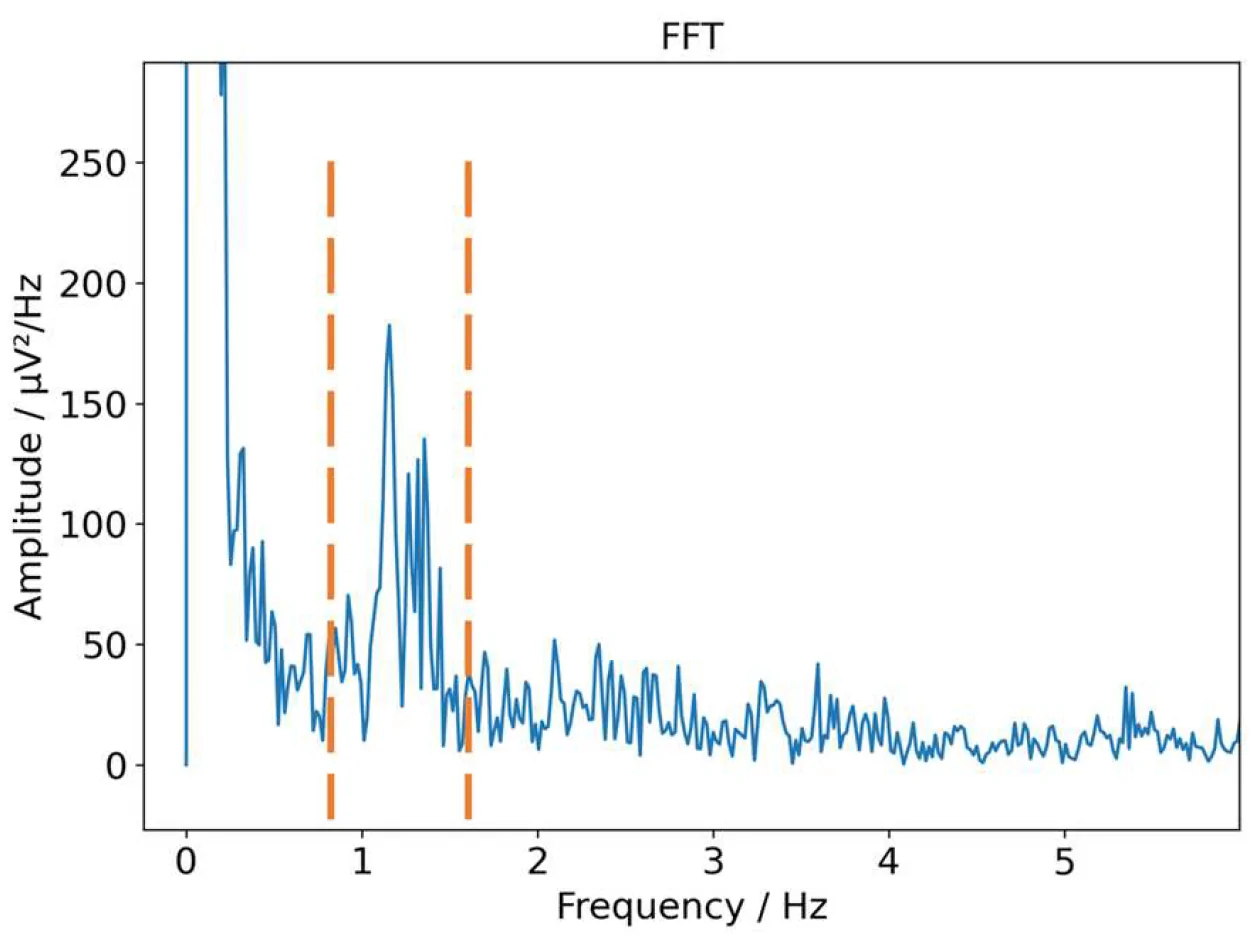
Frequency domain plot of fNIRS data during breath-hold and respiration.

Through the extinction coefficient graphs of oxyhemoglobin and deoxyhemoglobin at different wavelengths, we can see that the extinction coefficient of deoxyhemoglobin (HbR) is greater at an LED wavelength of 660 nm. Therefore, during breath-holding, oxyhemoglobin decreases and deoxyhemoglobin increases. The light emitted by the LED is absorbed more by deoxyhemoglobin, resulting in less scattered light in the brain. When breathing starts, oxyhemoglobin increases and deoxyhemoglobin decreases, which leads to more scattered light in the brain. The data presented in Figure 12 accurately reflects changes in blood oxygen in the brain from the time-domain graph. By analyzing the frequency-domain graph of the raw data, the spectral energy is more prominent between 1 Hz and 2 Hz. In the time-domain graph, a very small low-frequency peak can be seen above the original waveform, and we speculate that this may be the heart rate information reflected in the near-infrared data, as shown in Figure 13.

To verify our hypothesis, we used a PulseSensor (PulseSensor LLC) heart rate sensor to collect pulse rate data while measuring brain oxygenation and then calculated the correlation between the fNIRS data collected by our device and the data collected by the PulseSensor.

**Figure 14:**
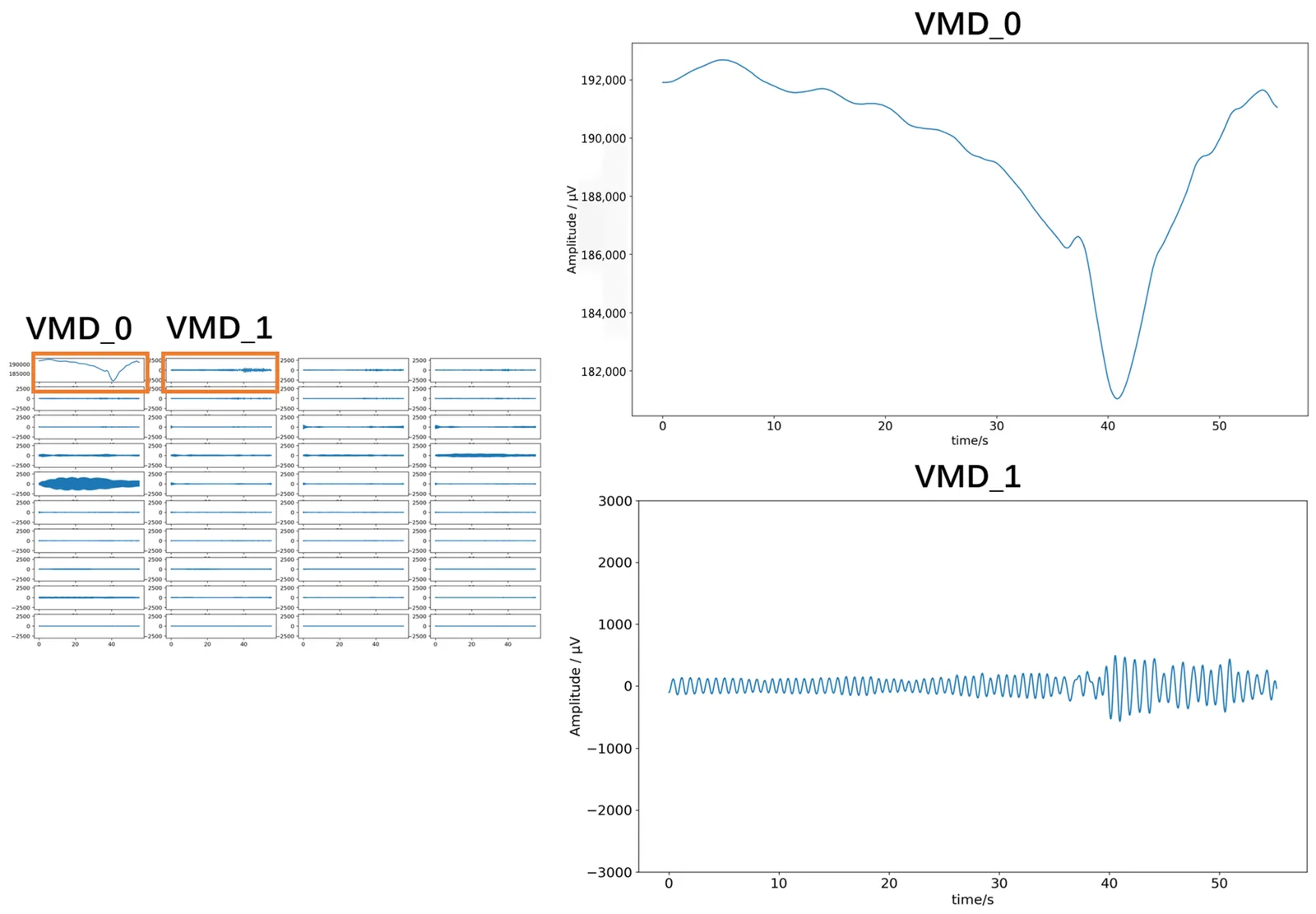
Signal presentation of different frequency components of fNIRS raw data decomposed by the variational mode decomposition algorithm.

Before performing correlation analysis, we need to preprocess the raw data and extract the heart rate signal from the original data. Variational mode decomposition (VMD) is an adaptive and completely non-recursive method for mode variation and signal processing [35]. In this method, the frequency center and bandwidth of each component are determined by iteratively searching for the optimal solution of the variational model during the process of obtaining the decomposition components. This allows for adaptive frequency domain segmentation of the signal and effective separation of each component. The left part of Figure 14 shows the components of the fNIRS raw signal after being decomposed using the VMD algorithm. In this algorithm, we set the number of decomposition modes to 40 and use plotting tools to display the various component signals generated by the algorithm. VMD_0 data represents the DC component of the fNIRS signal, which reflects changes in cerebral oxygen concentration. VMD_1 data is extracted from the original data and does not include data in the DC component or within the 0–3 Hz frequency band, where the frequency of the pulse rate signal is approximately centered.

Assuming we have a signal x(t), which can be represented as a linear combination of a set of local mode functions $\phi_{i}\left(t\right)$, i.e.,

$$x\left(t\right)=\sum_{i=1}^{K}\alpha_{i}\left(t\right)\phi_{i}\left(t\right)$$

where K is the number of modes we want to decompose.

Our goal is to find these mode functions $\phi_{i}(t)$ and their corresponding coefficients $\alpha_{i}(t)$. To achieve this, we define a cost function that represents the difference between the signal x(t) and the mode functions $\phi_{i}(t)$ and include some regularization terms to ensure that the decomposed modes have certain orthogonality and smoothness.

The cost function is defined as follows:

$$J\left(\alpha_{1},\ldots ,\alpha_{K},\phi_{1},\ldots ,\phi_{K}\right)\;=\sum_{i=1}^{K}\left[|x-\sum_{j=1}^{K}\alpha_{j}\phi_{j}{|}^{2}+\lambda |\nabla \alpha_{i}{|}^{2}+\mu |\nabla^{2}\phi_{i}{|}^{2}\right]$$

where ∥·∥ denotes a norm, λ and μ are regularization parameters, ∇ denotes the gradient operator, and $\nabla^{2}$ denotes the Laplacian operator. We aim to find a set of mode functions $\phi_{i}(t)$ and their corresponding coefficients $\alpha_{i}(t)$ such that the cost function J is minimized. By iteratively updating the mode functions $\phi_{i}(t)$ and coefficients $\alpha_{i}(t)$ until convergence, we can obtain the final VMD result.

In summary, the VMD algorithm optimizes a cost function to decompose a signal into a set of local mode functions and their corresponding coefficients. These mode functions effectively capture the oscillatory patterns and frequency components of the signal, allowing for signal decomposition and reconstruction.

**Figure 15:**
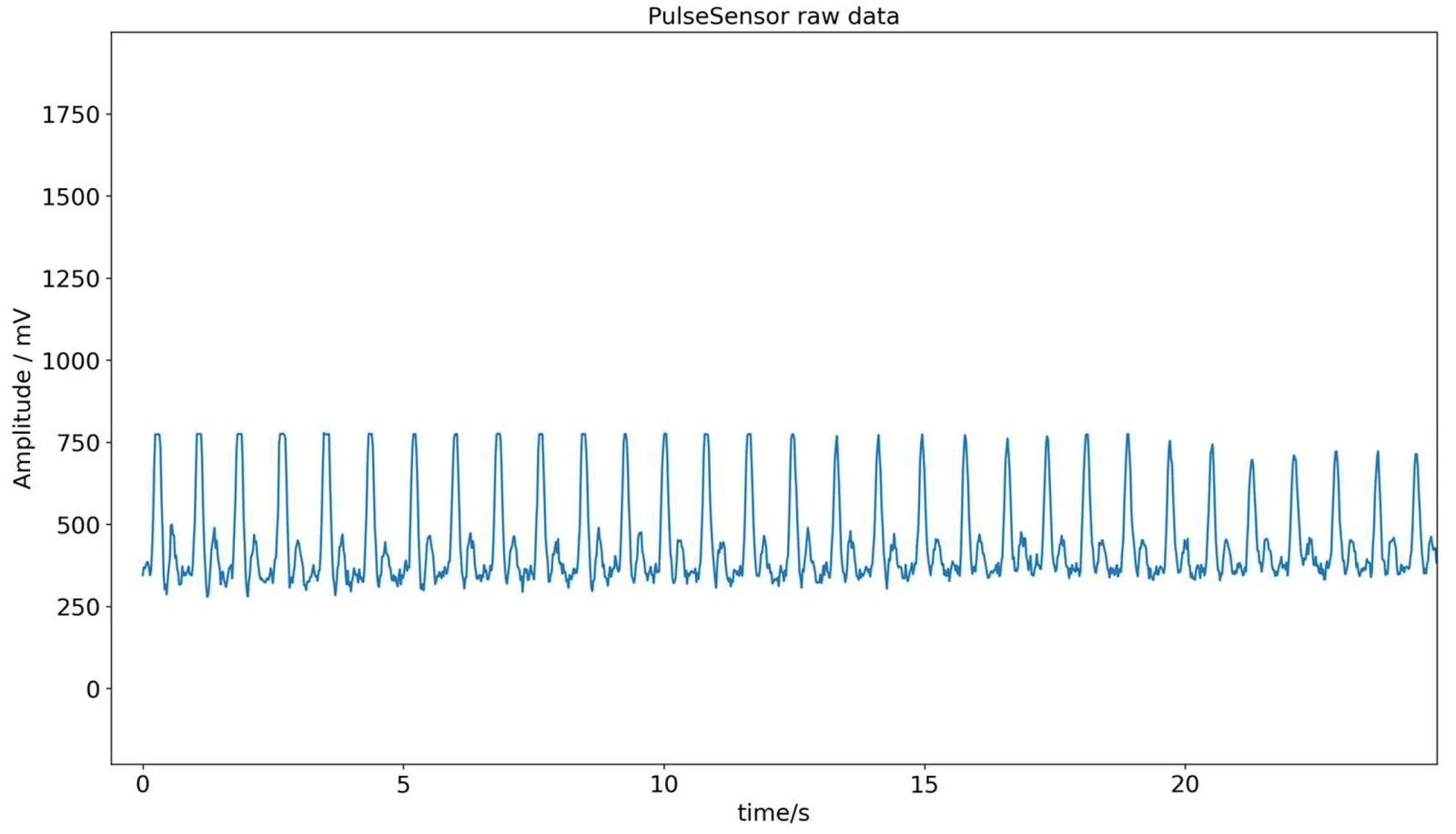
Raw data from PulseSensor.

Figure 15 shows the raw data of the pulse rate signal collected by PulseSensor, where each peak represents a diastole of the pulse and corresponds to the heart rate. The Pearson correlation coefficient, also known as the Pearson product-moment correlation coefficient, is a measure of the linear relationship between two random variables X and Y. Its formula is as follows, where Cov(X,Y) is the covariance between X and Y, Var[X] is the variance of X, and Var[Y] is the variance of Y. We use this formula to calculate the correlation between the fNIRS data collected by our device and the heart rate data collected by the PulseSensor.

(7) $$r\left(X,Y\right)=\frac{Cov\left(X,Y\right)}{\sqrt{Var\left[X\right]Var\left[Y\right]}}$$

The final calculation results are shown in Table 4. According to the calculation, the correlation coefficient between the 0–3 Hz band signal extracted by VMD from fNIRS data and the original data from the PulseSensor is 72.8%. After the original data from the PulseSensor is decomposed by VMD, the correlation coefficient between the two signals is 95%. This basically confirms that the fNIRS signal we collected contains heart rate information, reflects the richness of information in the fNIRS data, and indirectly confirms the effectiveness of our equipment. Analyzing the heart rate amplitude signal after VMD, it can be observed that the heart rate amplitude in the latter half is much larger than that in the first half, which corresponds to the subject’s heartbeat amplitude increasing when they start breathing rapidly after holding their breath for a long time.

### 3.4. Comprehensive Experiment

Depression is rampant globally, with significant impacts. A lot of research has been conducted in the laboratory using expensive and complex electroencephalogram (EEG) [27,28,29,36] and near-infrared equipment [37,38] for the diagnosis of depression. Participants with major depressive disorder were recruited from Ningbo Kangning Hospital. The study protocol was approved by the Scientific Research Ethics Review Committee of NingboTech University (approval no. LL2025080801; approval date: 8 August 2025). All the subjects passed the self-evaluation scale (PHQ-9) and the doctor’s diagnosis. Before participation, all participants were informed about the study procedures and provided written informed consent. All EEG and fNIRS data were collected under the condition of closed eyes and rest, which is more convenient and stress-free for the subjects than inducing emotions or other task paradigms. The frontal lobe three electrodes (Fp1, Fpz, Fp2) and corresponding near-infrared data are collected synchronously. The duration of the collection is 600 s, and the sampling frequency of near-infrared and EEG collection is 250 Hz. The entire experiment is conducted in a quiet room with low electromagnetic interference and good ventilation. A total of 31 independent subjects participated in this project, including 15 subjects with major depressive disorder and 16 healthy controls. The signal was first subjected to 50 Hz notch filtering, followed by bandpass filtering between 0.5 and 48 Hz, and removal of artifacts such as ocular and muscular electrical activity. Each 3 s segment of EEG and fNIRS signals was segmented from the original signals. After excluding contaminated segments, a total of 4239 samples were collected, including 2035 samples from the depressive subjects and 2204 from the healthy controls.

The literature shows that EEG rhythms are the embodiment of human emotions, and the corresponding frequency band energy can characterize the characteristics of emotions [39,40,41]. The 5th-order Butterworth filter bank was used to filter x(n) by sub-band, and the frequency bands were divided into B1 B6: [0.5–4 Hz], [4–8 Hz], [8–12 Hz], [12–18 Hz], [16–28 Hz], [21–48 Hz], and all frequency bands B0, i.e., [0.5 48 Hz], so as to obtain the energy characteristics of EEG signals in 7 frequency bands, a total of 7 characteristics.

Hjorth parameters are widely used in the feature analysis and processing of EEG signals because they can characterize the statistical characteristics of EEG signals from the time domain and have low computational cost. Activity is the variance of the signal, reflecting the average power of the EEG signal. Mobility represents the ratio of the root mean square of the slope of the EEG signal to the root mean square of the amplitude, which is a measure of the average frequency of the EEG signal and is used to estimate the average frequency. The mobility parameter is proportional to the standard deviation of the power spectrum, and the Complexity parameter gives an estimate of the signal bandwidth. Complexity calculates the root mean square of the ratio of the slope change in the signal to the ideal curve, which is used to measure the bandwidth of the EEG signal and can indicate the similarity of the EEG signal to a pure sine wave. Because the calculation of Hjorth parameters is based on variance, it is less computationally expensive than wavelet transform and short-time Fourier transform, which is suitable for real-time tasks. After the signal x(n) is decomposed into 7 frequency bands B0–B6 with the filter group, the three Hjorth parameters are obtained according to the following formulas, and the specific calculation is as follows, in which x(n) stands for the signal and the corresponding variance.

(8) $$\mathrm{Activity}\left(x\left(n\right)\right)=var\left(x\left(n\right)\right)$$

(9) $$Mobility\left(x\left(n\right)\right)=\sqrt{\frac{var\left(x^{\prime }\left(n\right)\right)}{var\left(x\left(n\right)\right)}}$$

(10) $$x^{\prime }\left(n\right)=x\left(n+1\right)-x\left(n\right)$$

(11) $$Complexity=\sqrt{\frac{Mobility\left(x^{\prime }\left(n\right)\right)}{Mobility\left(x\left(n\right)\right)}}$$

The Teager–Kaiser Operator (TKO) is a nonlinear energy operator that determines the instantaneous energy of a nonstationary signal, and in addition to the energy, the TKO can also track the instantaneous amplitude and frequency of the signal. The Hilbert transform (HT) can be used to separate instantaneous frequencies and amplitudes, but HT is applied to the global range, while local amplitude and frequency fluctuations can be effectively separated with TKO, which has better tracking capabilities for non-stationary signals such as EEG and near-infrared [42]. For signals x(n), TKO is defined as

(12) $$\psi \left(x\left(n\right)\right)=x^{2}\left(n\right)-x\left(n+1\right)\cdot x\left(n-1\right)$$

In the specific calculation, x(n) is first subjected to the discrete wavelet transform (DWT) of the following Equation (13). Applying wavelet decomposition, a four-level decomposition is performed to obtain the wavelet coefficients for different frequency bands. After obtaining the corresponding wavelet transform coefficients, the wavelet transform coefficients of each layer are processed according to the discrete TKO above as in Equation (12), and finally, the corresponding mean and variance are calculated as Teager–Kaiser features.

(13) $$DWT\left(m,n\right)=\int_{-\infty }^{\infty }x\left(t\right)\frac{1}{\sqrt{2^{m}}}\phi \left(\frac{t-2^{m}n}{2^{m}}\right)dt$$

**Figure 16:**
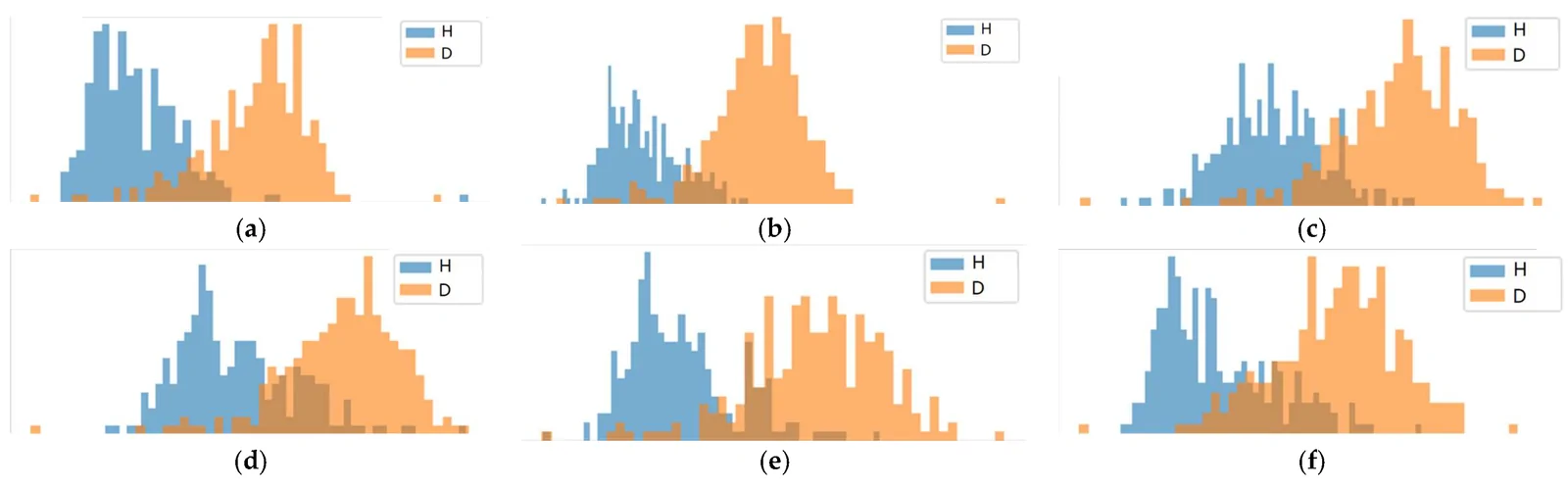
Statistical histogram of EEG and fNIRS features for healthy controls and depressive subjects. (**a**) Statistical histogram of alpha power of Fpz for healthy controls and depressive subjects; (**b**) statistical histogram of mobility of Fpz; (**c**) Statistical histogram of TKO energy of FP1; (**d**) statistical histogram of wavelet coefficient energy of Fpz; (**e**) statistical histogram of the mobility of HbO concentration; (**f**) statistical histogram of complexity of HbR concentration.

These features are selected and analyzed. Part of them is shown in Figure 16a–f below as statistical histogram comparisons of features for depressive subjects and healthy controls. We have applied a low-cost wearable device designed in this paper to collect EEG and fNIRS data from healthy and depressive individuals and carried out feature extraction as described above. Figure 16 represents the comparison of the statistical histograms of some features of the EEG and near-infrared data of the healthy controls and the depressive subjects. The *x*-axis shows the distribution of the calculated feature values, and the *y*-axis represents the probability distribution of each datum. H (blue color) denotes healthy controls, while D (orange color) denotes depressive subjects. As shown in Figure 16, various features extracted from EEG and near-infrared data, including band power, mobility, TKO energy, wavelet coefficient energy, etc., can all distinguish between healthy and depressive states with a significant probability. This further verifies the effectiveness and reliability of the signal collected by the designed circuit and provides a low-cost solution for rapidly diagnosing depression.

**Figure 17:**
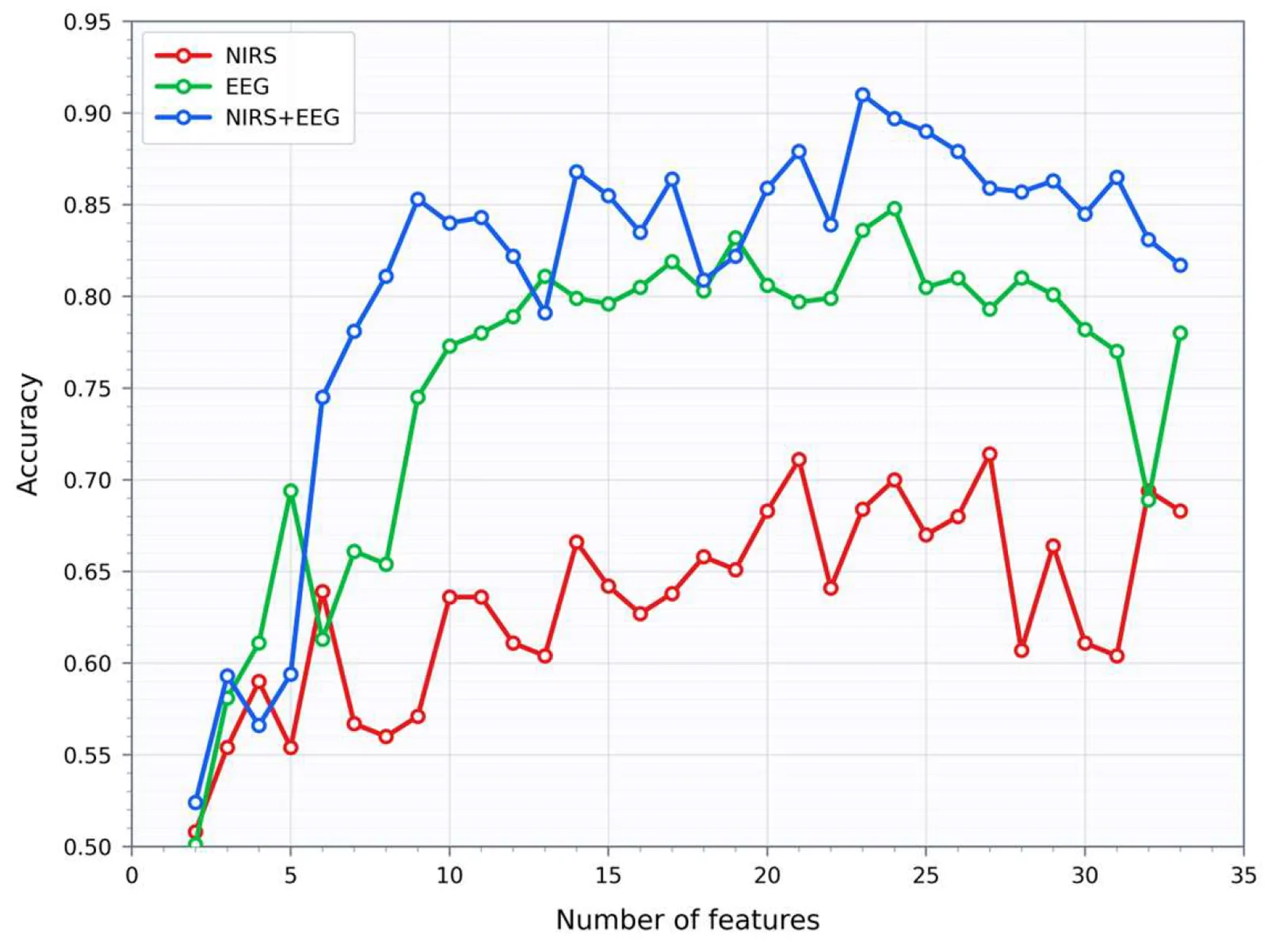
Comparison of classification performance of three modalities under different feature quantities.

To preliminarily verify the advantages of multimodal fusion of EEG and near-infrared spectroscopy (NIRS), three modal options (i.e., EEG, fNIRS, and EEG + fNIRS) were applied in the project. The selected features were compared in a classification experiment on the test set data, with different numbers of selected features. The classification accuracy is shown in Figure 17. The classification task is divided into two categories: depression and normal. A total of 4239 samples were collected from 31 participants. Five repeated stratified participant-wise splits were performed. In each split, 19 participants were assigned to the training set, including 9 participants with depression and 10 healthy controls; 4 participants were assigned to the validation set, including 2 participants with depression and 2 healthy controls; and the remaining 8 participants, including 4 participants with depression and 4 healthy controls, were used as the independent test set. Importantly, all segments belonging to the same participant were assigned to only one subset within each split. Therefore, no participant or segment from a test participant was used for model training, feature standardization, feature selection, or hyperparameter optimization. A support vector machine with a Gaussian radial basis function (RBF) kernel was used for classification. Before SVM training, each feature was standardized using z-score normalization based on the mean and standard deviation calculated from the training set. The same training-set parameters were then applied unchanged to the validation and test sets. Features were ranked using the Fisher score, and the number of retained features was selected according to the performance of the validation set rather than the independent test set. The regularization parameter C and kernel parameter γ were optimized using the validation participants over the predefined ranges of C = {0.01, 0.1, 1, 10, 100} and γ = {0.001, 0.01, 0.1, 1}. Once the optimal feature number and SVM parameters had been determined, the model was evaluated on the independent test participants without further parameter adjustment. The analysis was implemented in Python 3.10 using scikit-learn, and a fixed random seed of 100 was used. It can be seen that, first, the curve (classification accuracy) rises as the size of the best selected feature subset increases and then remains within the maximum accuracy range after increasing the number of features, reaching its maximum classification accuracy at a certain point before decreasing. Importantly, overall, the mixed EEG-fNIRS multimodal fusion performs better in classification accuracy than the other two single modalities. The revised participant-wise analysis showed that the highest mean test accuracies across the five splits were as in Table 5. These results were obtained using participant-disjoint training, validation, and test sets. It should be noted that the independent sample size was 31 participants, whereas the 4239 segments represented repeated signal observations. The preliminary experiment shows that EEG and NIRS have good distinguishing and complementary characteristics. The fusion of the two modalities can have better classification ability than a single modality.

All segments from the same participant were kept within the same subset to prevent data leakage; preprocessing and feature selection were fitted using only the training participants, while model and feature-number selection were restricted to the validation participants; segment-level SVM decision scores were aggregated into a single participant-level score, from which accuracy, sensitivity, specificity, F1 score, and AUC were then calculated. The participant-level results were as follows:

## 4. Discussion

This paper designs a portable, wearable EEG-fNIRS signal acquisition device. The device consists of two parts: electroencephalographic (EEG) acquisition and near-infrared (fNIRS) acquisition. It uses the STM32F103 chip from STMicroelectronics as the main MCU, the ADS1299 from TI as the analog front-end chip for the bio-signals, and a bidirectional LED driver circuit for the fNIRS light source to ensure stable light output. The device has 6 data channels (3 EEG + 3 fNIRS) with a sampling rate of 250 SPS and is equipped with a wireless module to send data wirelessly to a PC host computer.

## 5. Conclusions

In addition, an accelerometer MPU6050 has been added to the unit to filter out invalid data. The average internal noise of the system was measured to be below 3.13 µV, meeting the need for accurate acquisition of EEG and fNIRS data. Two representative experiments, the alpha wave rhythm experiment and the SSEVP experiment, were used to verify the functionality of the EEG part of the device, and the results demonstrated the effectiveness of the EEG acquisition. We verified the feasibility of fNIRS data acquisition by monitoring cerebral blood oxygen changes through breath-holding and exhalation experiments. The decrease and increase in cerebral blood oxygen concentration can be clearly seen in the fNIRS data. In addition, we also separated cerebral blood oxygen change data and heart rate data from the fNIRS raw data separately by using the variational mode decomposition VMD algorithm and did correlation analysis with the heart rate data. The results of this experiment also validated the validity of our fNIRS data acquisition. Furthermore, the proposed device was tested with 31 subjects (15 depressive subjects) to detect depression. Experiments proved the effectiveness of the multimodal signals, which outperformed single modal and surpassed EEG by 8.4% and fNIRS by 23.5%. The design in this paper has two advantages over conventional methods: (1) A lot of research has been conducted using EEG and near-infrared, but primarily using expensive and complex EEG and fNIRS equipment for signal collection and analysis [43,44,45]. Both EEG and fNIRS have significant advantages in cost and miniaturization, making them key to the development of wearable monitoring systems. Monitoring mental states such as depression often needs to be done over a long period to be more effective. (2) EEG and fNIRS are highly complementary, and their fusion has excellent application prospects [43,44,45]. EEG measures the activity and connectivity of neurons in the brain, while fNIRS measures neurovascular coupling, particularly the changes in concentration of oxygenated and deoxygenated hemoglobin. Both provide different perspectives on brain activity, enriching the measurement data and allowing for a deeper understanding of the brain activity characteristics of emotional and mental states. Additionally, EEG uses electrical signals, and fNIRS uses light signals, which do not interfere with each other, making simultaneous collection convenient [45]. Therefore, this paper designs a low-cost wearable circuit integrating EEG and fNIRS, enabling long-term monitoring and a deep grasp of users’ multi-dimensional brain activity data. Our experimental results also confirm the effectiveness of the designed wearable circuit and the superiority of multi-modalities.

## Figures and Tables

**Table 1:** According to the datasheets technical specifications and performance metrics.

Parameter	Value	Parameter	Value
Size	8.9 cm × 4.5 cm	System Noise	<1 μV
Weight	64 g	Light Source Wavelength	660 nm/905 nm
Power Input	3.3–5 V	Number of Channels	3 EEG + 3 fNIRS
Signal Acquisition	EEG + fNIRS	Portability	yes
Sampling Rate	250 Hz	Spatial Resolution	<3 cm
Minimum Resolution	0.16 μV	Battery Life	8–10 h

**Table 2:** Internal square wave signal error.

Gain	T = 1.024 s	T = 0.512 s
1	0.05%	0.06%
24	0.12%	0.12%

**Table 3:** Analog front-end chip noise testing.

Num Gain	1	2	3	4	Average (µV)
1	3.23	3.02	2.98	3.27	3.13
24	1.52	1.48	1.57	1.54	1.53

**Table 4:** Correlation analysis of heart rate data with fNIRS.

Subject	1	2	3	4	Average
fNIRS raw data	72.3%	73.4%	72.9%	72.6%	72.8%
VMD(0–3 hz)	95.2%	94.9%	94.6%	95.3%	95.0%

**Table 5:** Performances of different Modality.

Modality	Number of Features	Accuracy	Sensitivity	Specificity	F1 Score	AUC
fNIRS	27	72.5% ± 5.6%	75.0% ± 17.7%	70.0% ± 11.2%	72.4% ± 8.8%	0.763 ± 0.052
EEG	24	85.0% ± 5.6%	85.0% ± 13.7%	85.0% ± 13.7%	84.8% ± 5.7%	0.888 ± 0.052
EEG + fNIRS	23	90.0% ± 5.6%	90.0% ± 13.7%	90.0% ± 13.7%	89.8% ± 5.9%	0.950 ± 0.052

## Data Availability

The data that support the findings of this study are available from the corresponding author on reasonable request.
